# Nanocellulose: From Fundamentals to Advanced Applications

**DOI:** 10.3389/fchem.2020.00392

**Published:** 2020-05-06

**Authors:** Djalal Trache, Ahmed Fouzi Tarchoun, Mehdi Derradji, Tuan Sherwyn Hamidon, Nanang Masruchin, Nicolas Brosse, M. Hazwan Hussin

**Affiliations:** ^1^UER Procédés Energétiques, Ecole Militaire Polytechnique, Bordj El-Bahri, Algeria; ^2^Materials Technology Research Group, School of Chemical Sciences, Universiti Sains Malaysia, Penang, Malaysia; ^3^Research Center for Biomaterials, Indonesian Institute of Sciences (LIPI), Jakarta, Indonesia; ^4^Laboratoire d'Etude et de Recherche sur le MAtériau Bois (LERMAB), Faculté des Sciences et Techniques, Université de Lorraine, Vandœuvre-lès-Nancy, France

**Keywords:** nanocellulose, cellulose nanocrystals, production, functionalization, application

## Abstract

Over the past few years, nanocellulose (NC), cellulose in the form of nanostructures, has been proved to be one of the most prominent green materials of modern times. NC materials have gained growing interests owing to their attractive and excellent characteristics such as abundance, high aspect ratio, better mechanical properties, renewability, and biocompatibility. The abundant hydroxyl functional groups allow a wide range of functionalizations *via* chemical reactions, leading to developing various materials with tunable features. In this review, recent advances in the preparation, modification, and emerging application of nanocellulose, especially cellulose nanocrystals (CNCs), are described and discussed based on the analysis of the latest investigations (particularly for the reports of the past 3 years). We start with a concise background of cellulose, its structural organization as well as the nomenclature of cellulose nanomaterials for beginners in this field. Then, different experimental procedures for the production of nanocelluloses, their properties, and functionalization approaches were elaborated. Furthermore, a number of recent and emerging uses of nanocellulose in nanocomposites, Pickering emulsifiers, wood adhesives, wastewater treatment, as well as in new evolving biomedical applications are presented. Finally, the challenges and opportunities of NC-based emerging materials are discussed.

## Introduction

Nowadays, the application of green, renewable and sustainable materials has become increasingly important for producing various high-value products with low environmental impact (Oksman and Bismarck, 2014; Pandey et al., 2015; Thakur, 2015a,b; Kargarzadeh et al., 2018a,b). This area of research has attracted the interest of a great number of academicians and industrials as such materials turn out to be an alternative solution to the ever-depleting non-renewable sources, environmental pollution, global warming, and energy crisis. In this context, cellulose, starch, alginate, chitin, chitosan, and gelatin have been revealed to be promising candidates with regards to their abundant availability from various resources (Trache, 2018). Among them, cellulose is by far the most abundant renewable compound obtained from the biosphere and it can be found in plants, algae, tunicates, and some bacteria (Vazquez et al., 2015; Trache et al., 2016a,b). This fascinating polymer, seen as an inexhaustive source of raw materials, has potential to be modified and functionalized with several available industrial uses and there is still plenty to discover and celebrate in cellulose (Mokhena and John, 2020; Moohan et al., 2020; Trache et al., 2020). The benefit of cellulose can be further extended when cellulose chains are bundled together, generating highly ordered regions that can be subsequently isolated as nano-particles, known as cellulose nanomaterials or nanocelluloses, considered as useful class of futuristic materials (Foster et al., 2018) owing to their physicochemical features. In addition to be renewable and abundant, they combine chemical inertness, excellent stiffness, high strength, low coefficient of thermal expansion, low density, dimensional stability, and ability to modify its surface chemistry (Phanthong et al., 2018; Rajinipriya et al., 2018; Naz et al., 2019; Vineeth et al., 2019; Köse et al., 2020).

Typically, nanocellulose can be categorized into two major classes, (1) nanostructured materials (cellulose microcrystals and cellulose microfibrils) and (2) nanofibers (cellulose nanfibrils, cellulose nanocrystals, and bacterial cellulose) (Trache et al., 2017; Hussin et al., 2019; Pennells et al., 2020). A number of nanocellulose forms can be produced using different methods and from various cellulosic sources (Phanthong et al., 2018; Pires et al., 2019; Salimi et al., 2019). The morphology, size, and other characteristics of each nanocellulose class depend on the cellulose origin, the isolation and processing conditions as well as the possible pre- or post-treatments. The opportunity of producing nanocellulose with various features is considered fairly an exciting topic, which can promote the exploration of unexplored biomass. The benefits of the 3-D hierarchical nanostructure of nanocellulose and its physicochemical characteristics at nano scale open new prospects in several applications (Li et al., 2018; Vilarinho et al., 2018; Pires et al., 2019; Köse et al., 2020). According to Markets and Markets, the nanocellulose market is forecasted to achieve USD 783 Million by 2025. The rising demand and the employment of new applications have driven the researchers and the industry to exploit even more the employment of nanocellulose (Coelho et al., 2018). In addition, the number of papers is increasing year after year, reflecting the high concern in this type of nanomaterial. This attention expresses itself by the new International Organization for Standardization (ISO), Technical Association of the Pulp and Paper Industry (TAPPI), and Canadian Standards Association (CSA) Standards on CNCSs that are being developed and published, highlighting the market interest (Klemm et al., 2018). Nanocellulose, which can currently be produced in industrial scale at the tons per day, can be employed in several fields in our life, such as nanocomposite materials, biomedical products, wood adhesives, supercapacitors, template for electronic components, batteries, catalytic supports, electroactive polymers, continuous fibers and textiles, food coatings, barrier/separation membranes, antimicrobial films, paper products, cosmetic, cements, and many more emerging uses (Moon et al., 2016; Thomas et al., 2018).

The search of novel applications and improving the properties of the current nanocellulose-based materials are crucial driving forces for research and development (R&D) in various research groups and increasingly in companies. It can be seen that several literature review articles have been published during the last few years and most of them focused on the production of nanocelluloses, their modification and applications (Dufresne, 2019; He et al., 2019; Karimian et al., 2019; Kim J. H. et al., 2019; Luo et al., 2019; Miao and Hamad, 2019; Naz et al., 2019; Park et al., 2019; Salimi et al., 2019; Sharma et al., 2019; Shojaeiarani et al., 2019; Tan et al., 2019; Younas et al., 2019; Köse et al., 2020; Mokhena and John, 2020; Moohan et al., 2020; Tong et al., 2020). Certain recent findings and advances have not been enough addressed in previous publications, while here, we concisely provide some of the most recent applications of nanocellulose (NC), especially cellulose nanocrystals (CNC). The aims of this review is to make a brief summary on the study of nanocelluloses, with a special focus on CNCs, as well as their recent applications. At first, a brief introduction on cellulose, nanocellulose nomenclature, its isolation from several feedstocks, properties and functionalization are presented. Important challenges related to their production and new directions are addressed. In the subsequent sections, we shed light on current trends and recent research on the use of nanocellulose with special emphasis on nanocomposites, medical, Pickering emulsifiers, wood adhesives, adsorption, separation, decontamination, and filtration applications, to provide readers with a comprehensive overview of the advanced science and engineering of nanocellulose-based emerging materials and uses. Other emerging applications of nanocellulose such as papermaking, oil and gas drilling and cementing, energy storage systems, sensors and biosensors, which have been extensively reviewed in recent years (Du X. et al., 2017; Chen et al., 2018; Kim J. H. et al., 2019; Tayeb and Tayeb, 2019; Balea et al., 2020; Dai et al., 2020; Lasrado et al., 2020; Ramasamy and Amanullah, 2020; Zhang et al., 2020), are excluded and they are beyond the scope of the present review. It is expected that this review will forge new directions for the preparation of NC as well as the design and production of new NC-based materials for widespread advanced applications.

## Overview of Nanocellulose

### Structure and Source of Cellulose

**Figure 1:**
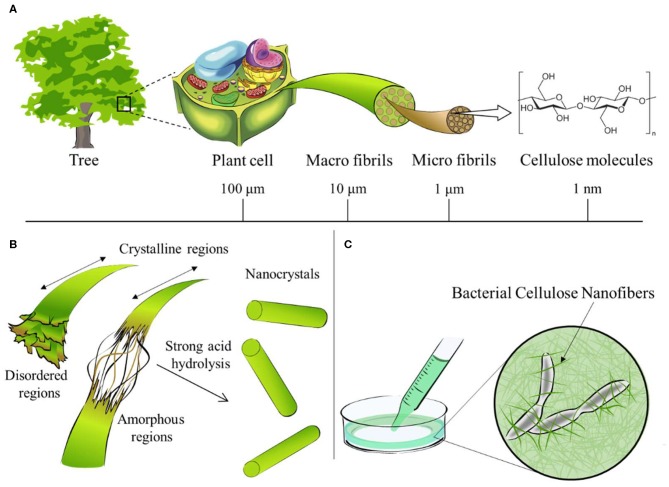
Cellulose contained in plants or trees has a hierarchical structure from the meter to the nanometer scale, as shown in **(A)**. A schematic diagram of the reaction between cellulose and strong acid to obtain Nanocellulose is shown in **(B)**. Bionanocellulose cultured from cellulose-synthesizing bacteria is shown in **(C)**. Reprinted with permission from Miyashiro et al. (2020) as distributed by creative common license CC BY license, MDPI publisher.

Cellulose, a fascinating and sustainable feedstock, is the most abundant polymeric raw material on earth. Its annual production is estimated to be between 10^10^ and 10^11^ t, but only a small portion of 6 × 10^9^ t is exploited by a number of industrial fields such as papers, textile, chemical, and material industries (Trache et al., 2020). Anselme Payen extracted this white biomacromolecule for the first time in 1838 and Herman Staudinger established its chemical structure few years later (Trache et al., 2016a). Cellulose is basically constituted by repeating β (1,4)-bound D-glucopyranosyl units (anhydroglucose unit, AGU) in the ^4^C_1_-chain configuration, in which every monomer unit is corkscrewed at 180° compared to its neighbors (Gopi et al., 2019). The generated cellobiose units are linked together to produce a crystalline structure of cellulose known as elementary fibrils. These latter are bundled together to produce micro-fibrils, which in turn formed macro-fibrils or cellulosic fibers. The intra- and intermolecular chemical groups impart cellulose its specific properties such as hydrophilicity, chirality, ease of chemical functionalization, insolubility in most aqueous solvents, and infusibility (Habibi et al., 2010). Obviously, cellulosic chains have a degree of polymerization of ~10 000 AGUs and 15 000 units, in wood- and cotton-derived cellulose, respectively. Cellulose characteristics are closely dependent on the degree of polymerization as well as the polymeric chain length. Native cellulose is composed of both ordered (crystalline) and disordered (amorphous) domains. Its crystallinity can vary from 40 to 70% depending on the natural source as well as the extraction procedure. The amorphous regions have low density compared to the crystalline ones and are prone to react with other molecular groups (Wertz et al., 2010; Dufresne and Belgacem, 2013; Kargarzadeh et al., 2017; Tarchoun et al., 2019a,b,c). Broadly, crystalline domains are more resistant to chemical, mechanical, and enzymatic treatments compared to the amorphous ones. Based on the molecular orientations, van der Waals, intra- and intermolecular interactions, isolation and treatment method, cellulose can be found as different polymorphs, i.e., cellulose I, II, III_I_, III_II_, IV_I_, and IV_II_, which can be transformed from one to another by using thermal or chemical treatments (Thakur, 2015a,b). Various sources (Table 1) such as wood, herbaceous plants, grass, agricultural crops and their by-products, animal, algae and bacterial sources, waste paper, among others, can be used as raw material to produce cellulose (Trache, 2017; Trache et al., 2017; Nandi and Guha, 2018; Kumar V. et al., 2020). A graphical presentation of cellulose from its natural raw material to the fundamental molecule is displayed in Figure 1. Cellulose with different features can be obtained depending on the natural source, its origin and maturity, pretreatment, and processing methodologies and reaction parameters (Dufresne, 2013; Zhao and Li, 2014; Ummartyotin and Manuspiya, 2015; Campano et al., 2016; Trache et al., 2017). Broadly, lignocellulosic sources require the elimination of non-cellulosic components through the removal of extractive components (fat, tannins, rein, rosin, free sugars, flavonoids, terpenoids, terpene, fatty acid, and waxes), the delignification and bleaching processes (Pires et al., 2019; Fodil Cherif et al., 2020). Such pre-treatments can be carried out using various chemical, physical, biological, and combined methods (Agbor et al., 2011; Karimi and Taherzadeh, 2016; Rabemanolontsoa and Saka, 2016; Hassan et al., 2018). They allow disrupting the compact structure of the lignocellulosic and overcoming its recalcitrance. Pretreatments present more than 40% of the total processing cost (Bhutto et al., 2017). The most important procedures can be found in several recent review papers (Behera et al., 2014; Karimi and Taherzadeh, 2016; Rabemanolontsoa and Saka, 2016; Bhutto et al., 2017; Hassan et al., 2018). These pretreatments allow the separation of pure and crystalline cellulose, ensure the break of the linkages existing between cellulose and non-cellulosic compounds (lignin and hemicellulose), decrease the degree of polymerization, promote the accessibility toward cellulose-rich fraction and increase the porosity, the inner surface and reactivity (Kargarzadeh et al., 2017). However, a number of pretreatments may negatively affect the process through the generation of toxic and hazardous wastes, imperfect separation, degradation, and loss of cellulose as well as of the high overall process expenses. For these reasons, several studied are still in progress over the world to well-understand the phenomena that can occur during the pretreatments, optimize the efficiency and the easiness of the processes, and reduce their costs and environmental impact (Phanthong et al., 2018). In the case of animal cellulose, some pretreatments are usually required to produce pure cellulose as reported by Trache et al. (2017). On the other hand, bacterial cellulose does not contain extractives, hemicellulose, and lignin with respect to vegetable cellulose, and thus does not necessitate specific pretreatments. Nevertheless, its production in industrial scale remains relatively expensive (Oun et al., 2020).

**Table 1:** Various sources for the production of cellulose fibers.

**Source group**	**Sources**
Hardwood	Eucalyptus, Aspen, Balsa, Oak, Elm, Maple, Birch
Softwood	Pine, Juniper, Spruce, Hemlock, Yew, Larch, Cedar
Annual plants/Agricultural residues	Oil palm, Hemp, Jute, Agave, Sisal, triticale straw, soybean straw, Alfa, Kenaf, Coconut husk, Begasse, Corn leaf, Sunflower, Bamboo Canola, Wheat, Rice, pineapple leaf and coir, Peanut shells, Potato peel, Tomato peel, Garlic straw residues, Mulberry fiber, Mengkuang leaves
Animal	Tunicates, *Chordata, Styela clava, Halocynthia roretzi Drasche*
Bacteria	*Gluconacetobacter„ Salmonella, Acetobacter, Azotobacter, Agrobacterium, Rhizobium, Alkaligenes, Aerobacter, Sarcina, Pseudomonas, Rhodobacter*
Algae	*Cladophora, Cystoseria myrica, Posidonia oceanica*

### Nomenclature and Types of Nanocellulose

Nanotechnology has become one of the driving forces behind a new industrial revolution in several fields, ranging from bionanocomposites, passing through medical, or even sensing and biosensing applications (Arof et al., 2019). Nanoscale materials have a size of about 100 nm in at least one dimension with specific physicochemical, optical, magnetic, and biological features compared to the bulk materials (He et al., 2019). Despite cellulose is widely studied for several decades, nanocellulose has emerged as a prominent and outstanding material in the last two decades, as indicated by three widely employed databases, namely Web of Science, PubMed, and ProQuest (Bacakova et al., 2019). This nanomaterial endows useful features such as high surface area-to-volume ratio, high Young's modulus and high tensile strength, low coefficient of thermal expansion, hydrogen-bonding capacity, biocompatibility, eco-friendliness, renewability, and lack of toxicity character (Salimi et al., 2019). The open literature has revealed that several terminologies have been and are currently used to define nanocellulose or cellulose nanomaterials, which unfortunately lead to misunderstanding and ambiguities (Trache et al., 2017; Hussin et al., 2019). Since anomalies still exist for nanocellulose nomenclature, it is important to introduce a standard nomenclature for the family of nanocelluloses, and efforts have to be devoted to rationalize the employment of the various terms based on their morphology, size, and synthetic procedures. Few years ago, the Technical Association of the Pulp and Paper Industry (TAPPI) has established a Nanotechnology Division dedicated to standardize the nomenclature of cellulose nanomaterials. A draft version standard, TAPPI WI 3021: Standard Terms and Their Definition for cellulose Nanomaterials, has been established (Dufresne, 2017; Kargarzadeh et al., 2018b). Broadly, nanocellulose can be categorized into nanostructured materials and nanofibers. The first category includes microcrystalline cellulose and cellulose microfibrils, whereas the second one comprises cellulose nanocrystals, cellulose nanofibrils, and bacterial cellulose. Cellulose nanocrystals (CNCs), usually produced by acid hydrolysis, consist of cylindrical, elongated, less flexible, and rod like nanoparticles with 4–70 nm in width, 100–6,000 nm in length, and 54–88% crystallinity index (Naz et al., 2019). It received a number of names throughout the two last decades encompassing nanocrystalline cellulose, rodlike cellulose crystals, nanowires, nanorods, nanoballs, cellulose crystallites, cellulose nanowhiskers, and cellulose whiskers (Brinchi et al., 2013; Charreau et al., 2013; Mariano et al., 2014; Vazquez et al., 2015; Trache et al., 2017). However, in the last few years the nomenclature has progressively converged to cellulose whiskers, cellulose nanowhiskers, and, more recently, to cellulose nanocrystals and nanocrystalline cellulose (Charreau et al., 2020). Nanofibrillated cellulose (CNF), commonly obtained by mechanical treatment, presents an entangled network structure with flexible, longer and wide nanofibers (20–100 nm in width and >10,000 nm in length), and lower crystallinity with respect to CNCs. Various names have been used for CNF such as cellulose nanofbril, nanofibrillar cellulose, and nanofibrous cellulose. The production of CNF from lignocellulosic biomass has most commonly performed through a range of chemical, mechanical, and enzymatic treatments, or a combination thereof, as recently described in detail elsewhere (Nechyporchuk et al., 2016; Osong et al., 2016). The common sources of CNF as well as its top research fields have been recently reviewed by Pennells et al. (2020). On the other hand, bacterial nanocellulose, also known as microbial nanocellulose, is considered as a promising and cost-effective natural nanomaterial for biomedical uses (Carvalho et al., 2019; Sharma and Bhardwaj, 2019). It consists of ultrafine, pure and ribbon-shaped nanofibers with 20–100 nm in diameter and micrometers lengths, which entangled to produce three-dimensional network as a hallmark. Such kind of nanocellulose is typically produced from bacteria, but its synthesis is seen extremely expensive because of the high costs of synthetic media (Trache, 2018). The amorphous nanocellulose (ANC) is another class of nanocellulose of spherical to elliptical shape with a diameter ranging from 80 to 120 nm. It can be prepared using acid hydrolysis with subsequent ultrasound disintegration from a regenerated cellulose, which can be obtained directly from cellulose solution via a physical dissolution, shaping, and regeneration process (Wang et al., 2016). ANC with enhanced properties such as high accessibility, improved sorption, and higher functional group amount can be primarily used as thickening agent in aqueous systems and carriers for bioactive substances (Kargarzadeh et al., 2017; Ram and Chauhan, 2018). Cellulose nanoyarn (CNY), one of the less investigated nanocellulose with diameters of 100–1,000 nm, is often obtained by electrospining of solutions containing cellulose or its derivatives. CNY finds application as wound dressings (Grumezescu, 2016). More recently, cellulose nanoplatelets (CNP), which are formed by entangled cellulose nanofibrils of 3 nm in diameter, have been prepared through oxidation under mild conditions. The thickness of such CNP is around 80 nm (Chávez-Guerrero et al., 2018).

The outstanding properties of cellulose nanofibers such as the nanometric scale, non-toxicity, high specific surface area, easy processing, high aspect ratio and stiffness, interesting mechanical characteristics, and good thermal stability have built up new opportunities for developing of novel kind of nanocellulose-based systems (Almeida et al., 2018; Dufresne, 2019; Liu et al., 2019b). Furthermore, many structures of nanocellulose, which can be prepared from various cellulosic sources based on several manufacturing methodologies, have permitted the development of different types of applications.

The emphasis of the following sections of this paper will be placed on one type of nanocellulose, i.e., cellulose nanocrystals, where the preparation methods, properties, surface modification as well as the recent applications of these nanomaterials will be treated. A special interest will be given to the studies carried out during the last 3 years and the term “nanocellulose” will be mainly reserved to describe CNCs, whereas only few details on CNFs are provided.

### Isolation Methods of Cellulose Nanocrystals

Despite being the most available biomacromolecule on the earth, only on the recent years that cellulose has received more attention as an outstanding nanomaterial for many applications and new added-values products. Owing to its nanoscale, nanocellulose exhibits various advantageous features than the bulk material, encompassing nanoscale effect, biocompatibility, biodegradability, high specific surface area, high crystallinity, purity, amphiphilic nature, surface chemical reactivity, barrier properties, high mechanical strength, green and non-toxic (Chen et al., 2018; Klemm et al., 2018; Nascimento et al., 2018). However, depending on the natural source, isolation procedure, conditions, and pre-post-treatments, the characteristics of nanocellulose such as crystallinity, yield, dimensions and morphology, surface chemistry, physicochemical, and thermal properties can be tailored for a specific use, opening an extensive range of possibilities to develop new materials and devices (Wang Z. et al., 2017; Phanthong et al., 2018; Thomas et al., 2018; Wohlhauser et al., 2018). They can be employed in energy storage, substrate for printing electronics, aerogels, emulsion stabilizers, support for catalysts and immobilization of enzymes, low-calorie food additives, templates, reinforcing polymer composite, liquid crystals, pharmaceutical binder, biomimetic materials, biosensors and bio-imaging, etc. (Serpa et al., 2016; Agate et al., 2018; Seabra et al., 2018; Dufresne, 2019; Kim J. H. et al., 2019).

The preparation of nanocellulose from cellulose requires typically two main stages (Trache et al., 2017; Nandi and Guha, 2018; Xie et al., 2018). The first, as summarized in Table 2 (See also the above section: Structure and source of cellulose), focuses on the pretreatments of feedstocks to obtain pure cellulose, whereas the second stage is dedicated to the transformation of cellulose to nanocellulose. During the first stage, extractives (monomers, dimers and polymers of fat, free sugar, tannins, resin, rosin, flavonoids, terpenoids, terpene, waxes, fatty acids, etc.), hemicelluloses and lignin have to be partially or totally eliminated from the feedstocks based on specific pre-treatment methods (Kargarzadeh et al., 2017; Chen et al., 2018). The second stage, however, is usually dedicated to the production of cellulose nanocrystals. This latter ensures the elimination of amorphous domains form pristine cellulose, giving rise to the production of CNCs (Dufresne, 2013, 2017; Jonoobi et al., 2015). The disordered regions distributed as chain dislocations on segments along the elementary fibril are prone to hydrolytic action because of the reduced steric hindrance and kinetic factors, whereas the ordered domains, which present a higher resistance to the hydrolysis process, remain intact. Subsequently, the cellulose fibrils are transversely cleaved, generating the short CNCs with relatively high crystallinity. Nevertheless, after this second stage, further post-treatments such as solvent elimination, neutralization, washing, purification, filtration, centrifugation, sonication, dialysis, fractionation, surface modification, stabilization, and drying (freeze-drying, spray-drying) need to be undertaken after the hydrolysis process to recuperate CNC product.

**Table 2:** The most employed and/or recently explored pre-treatment processes of lignocellulosic biomass, their advantages and shortcomings.

**Category**	**Pre-treatment process**	**Advantages**	**Shortcomings**	**References**
Chemical	Diluted acid	- Low acid consumption. - Extensive hemicellulose degradation.	- Corrosion of the equipment but low temperature is necessitated. - Degradation of cellulose. - Inhibitor formation increases with increase in temperature.	Agbor et al., 2011; Rabemanolontsoa and Saka, 2016; Bhutto et al., 2017; Chen et al., 2017; Hassan et al., 2018; Satlewal et al., 2018
	Concentrated acid	- Extensive hemicellulose degradation.	- Corrosion of the equipment, toxicity of the environment, requires high amount of acid, and energy consumption for acid recovery. - Degradation of cellulose. - Inhibitor formation increases with increase in concentration.	
	Organic acids	- Avoid equipment corrosion, low energy consumption for acid recovery.	- Less efficient for biomass with higher hemicellulose content.	
	Alkaline	- Disrupts the lignin structure. - Removes acetyl groups from hemicellulose. - Mild reaction conditions.	- Long residence time. - Neutralization issues.	
	Ionic liquids (ILs)	- Efficient lignin elimination. - Mild reaction conditions. - Some of them are reusable. - Better thermal stability.	- Commercial application requires more implementation to overcome the scaling challenges. - The challenge of polysaccharides recovery. - High price of chemical products. - Cellulose degradation.	
	Deep eutectic solvents (DES)	- Easy to prepare, stable, cost-effective, and most of them are environmental-friendly. - Mild reaction conditions. - Efficient dissolution of lignin. Disrupts lignin-carbohydrate complexes.	- Its efficiency depends on the nature of biomass. - Present high viscosity. - DES need to be manufactured at an industrial scale for availability as low-cost green solvents.	
	Oxidation	- Elimination of hemicellulose and lignin.		
	Organosolv	- Selective pretreatment methodology generating three separate fractions: dry lignin, relatively pure cellulose fraction, and an aqueous hemicellulose stream. - Low boiling point organic solvents are always easy to recover by distillation. - Efficient biomass delignification. - Increase the surface area. - Lack of toxicity, low price, and ease of recovery.	- Formation of inhibitor during lignin dissolution. - Required high pressure during pretreatment.	
Physical	Mechanical splintered	- Increase the specific surface area and decrease the particle size, which improve the hydrolysis yield.	- Requires more energy for hardwood than agricultural residues. - Less efficient process.	Singh R. et al., 2014; Bhutto et al., 2017; Rodriguez et al., 2017; Hassan et al., 2018; Liu et al., 2019a; Rezania et al., 2020
	High-intensity ultrasonication	- Lignocellulosic biomass is commonly treated by ultrasound acoustic wave with the frequency range from 10 kHz to 20 MHz. - The generated cavitations, which depend on the frequency, will collapse and release huge amount of energy that create a localized hot spots at temperature of 2,000–5,000°C and pressure of 500–1,800 bars with a life time of a few microseconds. - No toxic with reduced reaction time. - Degrades preferentially the lignin. - Decreases hemicellulose content.	- Depends closely on the nature of biomass and experimental conditions. - The efficiency and reliability of the operation depend on the ultrasonic mode (continuous or pulse), frequency, power, processing temperature, solvent, aeration, and the design of reactors with proper geometric construction.	
	Microwave radiation	- The process can be carried out at temperature of 50 to 210°C for 5–25 min. - A non-ionizing microwave radiation with a wavelengths ranging from 1 mm to 1m has frequency of 300 to 300,000 Mhz.		
		- Higher microwave power with short pretreatment time and the low microwave power with long pretreatment time had almost same effect. - The process insures fast heat transfer, short duration time, selectivity and uniform volumetric heating performance, easy operating and energy efficient. - Green technology. - Fast fractionation and lignin disruption. - Degrade hemicellulose.	- Increase the degradation of cellulose.	
	Gamma radiation	- Gamma radiation, obtained fromradioisotopes (Cobalt-60 or Cesium-137) can easily penetrate the lignocellulosic structure. - The most effective irradiation doses (891–1,200 kGy) possesses the most efficiency as ILs pretreatment. - Improves the post-treatments efficiency.	- May cause the cellulose degradation.	
Physicochemical	Wet oxidation	–The process requires treatment with water and air or oxygen at temperatures above 120°C under pressure up to 20 MPa for a period time 5–120 min. - Economic and available. - Assists to hemicellulose hydrolysis.	- Costs may be high.	Singh R. et al., 2014; Karimi and Taherzadeh, 2016; Bhutto et al., 2017; Chen et al., 2017; Hassan et al., 2018; Rezania et al., 2020; Zhao et al., 2020
	Hydrothermolysis	- The process can be carried out at temperature of 140 to 220°C for 4–180 min. - Environmentally friendly. - No corrosion problems. - Effectively removes hemicellulose. - Reduces the need for post-treatments.	- More energy demanding.	
	Steam explosion	- Biomass is treated with hot steam at 180–240°C under pressure (1–3.5 MPa) to improve the hemicellulose hydrolysis and the depolymerization of lignin, which are than enhanced with the second stage of depressurization. - Cost-effective process and low energy consuming. - Disruption of lignin and hemicellulose. - Can be used at commercial scale. - Less energy consumption and cost effective.	- Incomplete disruption of lignin-carbohydrate matrix. - Generation of inhibitors that can affect the post-processing. - Requires high pressure. - Excessive cellulose degradation.	
	Supercritical fluid	- Moderate critical temperature of 31.1°C and pressure of 7.4 MPa, and high solid capacity. - CO_2_ is inert in nature, inexpensive, non-toxic, non-inflammable, and available from the by-products of several industrial processes. - No generation of toxins. - Green technology and readily available.	- Less treatment efficiency. - High costs.	
	Ammonia fiber explosion (AFEX)	- AFEX is a dry-to-dry process. No wash stream in the process, and no toxic chemicals are produced for downstream processes. - Requires moderate temperature (<100°C), pH (<12), and short time duration. - Ammonia is the common chemical. - Insure the cleavage of lignin-carbohydrate complex linkages as well as the C-O-C bonds in lignin.	- Less effective for biomass containing high lignin content. - Requires high energy input for recycling and recovery.	
	Ammonia recycle percolation (APR)	- In APR process, the aqueous ammonia (10–15 wt%) pass through biomass at elevated temperature (150–170°C). Lower temperature (80–150°C) was also reported. The residence time varies between 5 and 30 min. - Efficient treatment for hardwood and herbaceous plants.		
		- Recyclable. - Causes the depolymerization of lignin and cleavage of lignin-carbohydrates linkages. - Relatively low cost process.	- Less effective for softwoods.	
Biological	Microbial	- Elimination of lignin and hemicellulose. - Low energy consumption. - Mild reaction conditions - No release of hazardous and harmful compounds.	- Relatively time consuming processes. - Some additional pre-treatments may increase costs. - Require large space and specific growth conditions. - Require further research activities to understand some important parameters such as kinetics.	Behera et al., 2014; Singh R. et al., 2014; Chen et al., 2017
	Fungal species			
	Enzymatic			
	Consolidated bioprocessing			
Combined	At least two of the above-mentioned pre-treatment processes	- Increase the efficiency of the elimination of lignin and hemicellulose at reasonable time.	- May enhance the operating cost. - Require further research work to optimize the processes. - Balance needs to be struck between efficiency improvement and cost.	Chen et al., 2017; Liu et al., 2019a

**Figure 2:**
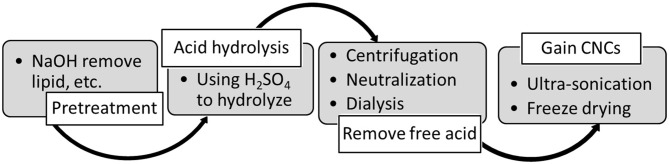
An example of the procedure used to prepare CNC form hardwood. Reprinted with permission from Lin K. H. et al. (2019) as distributed by creative common license CC BY license, MDPI publisher.

Despite acid hydrolysis using sulfuric acid is the oldest process, it remains the most common preparation method of CNCs. A typical approach starts with alkali and bleaching pretreatments followed by acid hydrolysis. It was reported that Calvert was the first author who performed the hydrolysis of cellulose in 1855 (Mao et al., 2017). Few decades later, in 1951, Rånby has successfully prepared stable colloidal suspensions of cellulose using H_2_SO_4_ (Nascimento et al., 2018). Nonetheless, the presence of sulfate esters at the cellulose surface decreases its thermal stability, but permits a well-dispersion of individual CNC bundles in aqueous media. An example of the procedure used to prepare CNCs form hardwood is depicted in Figure 2.

In recent years, hydrochloric, phosphoric, and hydrobromic either liquid, solid or gaseous acids, organic acids, or mixtures of inorganic and organic acids have been used to produce CNC (Trache et al., 2017). For instance, the use of hydrochloric acid instead of sulfuric acid for cellulose hydrolysis will generate CNCs with better thermal stability, but the lack of electrostatic repulsion force between crystals causes them agglomeration and less stable aqueous solution can be obtained (Gopi et al., 2019). Thus, both of reaction conditions and cellulose origin affect the properties of the obtained CNCs such as the morphology, aspect ratio, density, mechanical features, thermal stability, dimensional dispersity, and crystallinity.

In the pursuit of lowering production costs, improving the characteristics of nanocellulose, several preparation methods of CNCs have been published and others continue to be developed worldwide, seeking an economic/sustainable approach to produce CNCs with preferred features. A number of processes have been reported to obtain CNCs, namely, improved chemical acid hydrolysis (Thakur, 2015b; Du et al., 2016; Kontturi et al., 2016), mechanical treatment (Pandey et al., 2015), oxidation methods (Sun et al., 2015; Vazquez et al., 2015), enzymatic hydrolysis (Anderson et al., 2014; Tong et al., 2020), ionic liquid treatments (Lazko et al., 2016), deep eutectic solvents (Sirviö et al., 2016), subcritical water hydrolysis (Novo et al., 2015, 2016), and combined processes (Trache et al., 2017, 2020; Xie et al., 2018). This latter class seems to be an interesting path since these methods combine two or many processes, which can overcome the shortcomings of the single approaches by improving the CNC properties, increasing the yield and reducing the cost. Some recent approaches to produce CNCs are displayed in Table 3. Trache et al. have recently reported the advantages and shortcomings of some important hydrolysis processes (Trache et al., 2017, 2020). Despite the aforesaid reports, there are some potential concerns associated with CNCs preparations. Broadly, their isolation is time consuming, high energy-demanding and requires more attention to avoid the use of toxic chemicals, which are detrimental to human and the environment.

**Table 3:** A selection of recent CNC production methods from the corresponding natural source.

**Natural source**	**Methodology**	**References**
Filter paper and microcrystalline cellulose	Solution plasma-chemical processing as an oxidation–hydrolysis strategy	Surov et al., 2018
Cotton linters	Single step ammonium persulfate-assisted swelling, followed by oxidation	Wang et al., 2019
Cellulose fibers	Ball mill assisted fully recyclable solid acid hydrolysis	Song et al., 2018
Broomcorn Stalks	Acid hydrolysis followed by sonication	Langari et al., 2019
Eucalyptus hardwood	Irradiation oxidation and organosolv solubilization	Zhang and Liu, 2018
Microcrystalline cellulose	Ultrasonic pretreatment in ionic liquid followed by acid hydrolysis	Pang et al., 2018
Nata de coco	Ultrasonic irradiation coupled with microwave treatment	Wardhono et al., 2018
Oil palm	Sono-assisted TEMPO oxidation	Rohaizu and Wanrosli, 2017
Wood sawdust	Sono-chemical synthesis using acid hydrolysis	Shaheen and Emam, 2018
Microcrystalline cellulose	Recyclable citric/hydrochloric acids	Yu et al., 2019
Commercial microcrystalline cellulose	Facile and rapid one-step hydrolysis by H_2_SO_4_/HNO_3_ mixed acid	Cheng et al., 2020
Blue agave leaves and bagasse fibers	Sonochemical acid hydrolysis enhanced with sonication	Robles et al., 2018
Eucalyptus pulp	Periodate oxidation route followed by reductive treatment with NaBH_4_	Errokh et al., 2018
Cotton cellulose powder	High-pressure homogenization controlling a process temperature	Park et al., 2019
Commercial microcrystalline cellulose	A two-step collaborative process combining solvothermal pretreatment and mechanical exfoliation	Gao et al., 2019
Commercial microcrystalline cellulose	Ball milling with water followed by centrifugation	Kang et al., 2018
Lignocellulosic biomass	Hydrolysis by Ni(II)-transition metal salt followed by washing with distilled water, centrifugation, sonication and dialysis	Yahya et al., 2018

More recently, Charreau et al. have emphasized the increasing industrial interest on the field of cellulosic nanomaterials, which is evidenced by the astonishing increase in nanocellulose patents since 2010, and especially within the last 5 years, suggesting that the increasing trend would not stop in the following years (Charreau et al., 2020). For instance, more than 950 documents refereeing to CNCs have been published from 2010 and 2017. Most of them refer to the isolation methods, derivatization techniques as well as to different products containing these particles. However, it is worthy to note that the technology transfer, i.e., scaling-up form laboratory to bulk-scale is one of the major problem (Mishra et al., 2019). Overall, some methods are shorter and others are longer, some are environmentally benign whereas others are not, some are economic and less effective while others are efficient but expensive. Therefore, the journey so far not been so worthwhile. Nonetheless, more efforts are being devoted on the path to surmount all present-day obstacles. Besides that, some commercial producers currently prepare CNCs at capacities beyond pilot plant scale such as CelluForce (Canada, 1,000 kg/day), American Process Inc. (USA, 500 kg/day), Melodea/Holmen (Sweden, 100 kg/day), Blue Goose Biorefineries (Canada, 10 kg/day), Alberta Innovates (Canada, 100 kg/week), US forest products lab (USA, 10 kg/day), India Council for Agricultural Research (India, 10 kg/day), FPInnovation (Canada, 3 kg/day) (Xie et al., 2018; Trache et al., 2020). Nonetheless, the utilization of alternative cellulose sources to produce large-scale of CNCs remains timid. In this sense, the price of these nanomaterials is expected to decrease with the employment of cheaper sources of pulps and the optimization of extraction process. Moreover, The increase of the production rate worldwide, in the years to come as forecasted could bring down the cost significantly as well.

## Properties and Surface Modification of Nanocellulose

### Characterization and Properties of Nanocellulose

Depending on the source or origin (mainly from higher plants, algae, and bacteria), cellulose consists of varying portions of mostly crystalline (highly ordered) regions accompanied by some amorphous (disordered) fractions (George and Sabapathi, 2015). Upon isolating the crystalline regions from the biomass, it results in attaining polysaccharide nanocrystals, most commonly in the form of rod-shaped cellulose nanocrystals (Lin et al., 2012). Cellulose, the most ubiquitous biopolymer, in the form of nanocellulose (NC) has gained growing interest among researchers corroborating to its mechanical, physicochemical, and biological properties in consort with being eco-friendly (Saba et al., 2017). Nanocellulose (NC) can be obtained from natural cellulose, with few to tens of nanometers size range at least in one dimension. Xu et al. systematically discussed on nanocrystalline cellulose suspensions in the perspective of rheology, liquid crystal ordering, and colloidal phase behavior (Xu Y. et al., 2019). It was pointed out that geometrical dimensions and the morphology of cellulose nanocrystals vary based on their origin, extraction methods, and manufacturing conditions, which causes inconsistencies in suspension rheology and colloidal behaviors. The authors concluded that the rheology and colloidal behavior of aqueous nanocrystalline cellulose suspensions are comprehensively explained by colloidal volume fraction, the dimension of nanocrystalline cellulose rods and interparticle forces. Moreover, nanocrystalline cellulose suspensions form an ordered liquid crystal state when its concentration reaches a critical value. Abitbol et al. deduced that the stability of cellulose nanocrystal suspensions in water could be amended by regulating the surface charge, i.e., the degree of substitution of sulfate groups on their surface (Abitbol et al., 2018). The study presented that the viscosity of nanocellulose suspensions was inclined by surface charge, where CNCs with lower surface charge forms more viscous suspensions, consequently undergo gelation at lower concentrations. Researchers concluded that the effective volume of suspensions plays a major role throughout the concentration range relevant to liquid crystalline phase formation once the surface charge density of CNCs reaches a threshold value.

**Figure 3:**
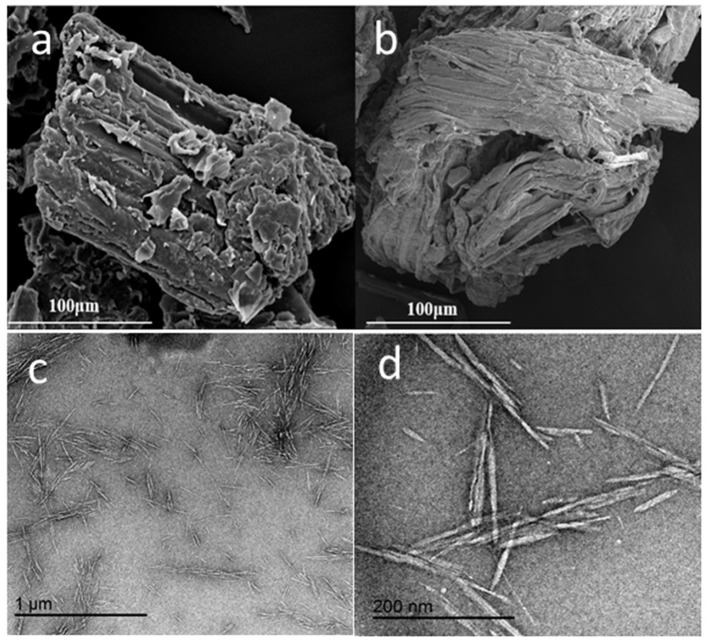
SEM micrographs of **(a)** the raw material (corn stalk) and **(b)** the extracted cellulose; **(c,d)** transmission electron microscopy (TEM) micrographs of the cellulose nanocrystals (CNCs). Reprinted from Huang et al. (2017) as distributed by creative common license CC BY license, MDPI publisher.

**Figure 4:**
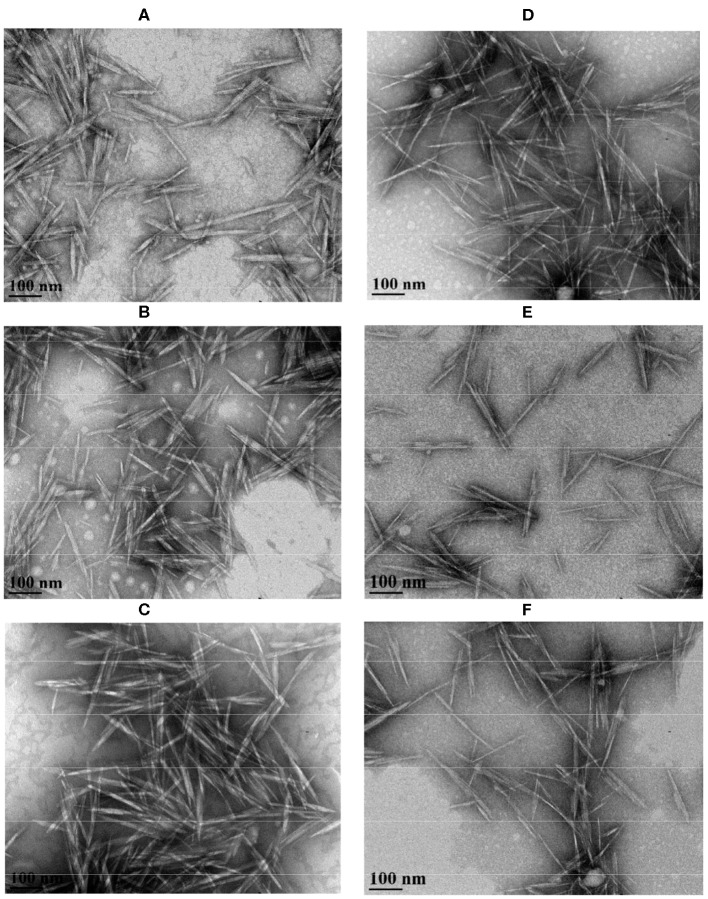
Transmission electron microscopy (TEM) micrographs of CNC isolated from: filter paper enzyme-treated for **(A)** 0 h, **(B)** 2 h, and **(C)** 10 h and wood pulp enzyme-treated for **(D)** 0 h, **(E)** 2 h, and **(F)** 10 h. Reprinted from Beyene et al. (2018) as distributed by creative common license CC BY license, MDPI publisher.

Nanocellulosic materials can be characterized by employing various techniques for instance; nitrogen gas and water adsorption isotherms, X-ray diffraction (XRD), helium pycnometry, dielectric spectroscopy, and mechanical testing to infer their properties (Le Bras et al., 2015). The crystallinity index (crystallinity percentage), which governs the mechanical and physical properties of nanocellulose is calculated employing an X-ray diffractometer, based on the maximum intensity of diffraction of the (002) lattice peak representing crytsalline region (I002) and the intensity of diffraction between (002) and (101) lattice peaks representing amorphous region (Iam). Thus, XRD peak position (2θ) and their intensities are referred in order to determine crystallinity. Morphological and dimensional evaluation of nanocellulose can be carried out using transmission electron microscopy (TEM), field emission scanning electron microscopy (FESEM), and atomic force microscopy (AFM) (Mohaiyiddin et al., 2016; Foster et al., 2018). Some TEM micrographs of CNCs prepared from different sources are displayed in Figures 3, 4, showing that their particle size ranges from 70 to 300 nm in length and 4 to 20 nm in width.

Density and the porosity of nanocellulose are measured using a helium pycnometer. Specific surface area and total pore volume can be obtained through the characterization involving N2 sorption analysis (Le Bras et al., 2015). Yang et al. (2017) reported that the zeta potential value, which examines the surface charge, reflects the dispersion stability of nanocellulose. Elemental composition on the surface of nanocellulose samples can be analyzed via X-ray photoelectron spectroscopy (XPS), while thermal stability, which signifies the thermal decomposition and impurities of nanocellulose, can be investigated through differential scanning calorimetry (DSC), thermogravimetric analysis (TGA), and differential thermogravimetry (DTG) (Jordan et al., 2019).

Utilization of dynamic nuclear polarization (DNP)-enhanced NMR spectroscopy has been an emerging tool to comprehend the surface chemistry of cellulose as it enhances conventional NMR sensitivity by several orders of magnitude (Smith et al., 2019). In a most recent study (Kumar A. et al., 2020), high-field dynamic nuclear polarization enhanced solid-state NMR technique has been employed to characterize surface species of metronidazole drug-functionalized TEMPO-oxidized cellulose nanofibrils, where comprehensive structural and chemical characterization of nanocellulose surface chemistry is still limited, especially for a very low level of functionalization (<1 wt.%). The researchers grafted the metronidazole drug onto CNF through a Diels-Alder reaction under heterogeneous aqueous conditions. Dynamic nuclear polarization enhanced-NMR data explicitly accounted the presence of trace amounts of TEMPO components and depolymerized cellulosic units in CNF, as well as coupling agents (1-Ethyl-3-(3-dimethylaminopropyl)carbodiimide and N-hydroxysuccinimide) on its surface. Moreover, their study showed that DNP-enhanced NMR spectroscopy is the only technique that can differentiate between surface adsorption and grafting.

With regards to cellulose's natural nano-structure, various methods can be employed to discover the characteristics of its structure through obtaining three different forms of nanocellulose such as cellulose nanocrystals (CNC), otherwise known as whiskers or microcrystallites, micro-fibrillated cellulose (MFC), also denoted as cellulose nanofiber (CNF), or nano-fibrillated cellulose (NFC) (Kaboorani and Riedl, 2015), and bacterial nanocellulose (BNC) (Gao et al., 2020). These three different forms of nanocellulose vary in their morphology (Liu et al., 2016). The isolation method governs the morphology and properties of nanocellulose (Yang et al., 2017). Nanocellulose exhibits some exclusive features such as exceptional mechanical properties (i.e., low density, high flexibility, and strength while being chemically inert) (Lavoine and Bergström, 2017) and thermal properties (Gan et al., 2020). Over the past few decades, many research studies have been conducted on the reinforcement of polymer matrix nanocomposites, for instance, natural rubber nanocomposites (Neto et al., 2016; Cao et al., 2018; Dominic et al., 2020), polylactic acid nanocomposites (Gitari et al., 2019; Rigotti et al., 2019), epoxy nanocomposites (Ayrilmis et al., 2019; Yan et al., 2019; Yue et al., 2019), and polystyrene nanocomposites (Clarke et al., 2019; Neves et al., 2019), where nanocellulose has been introduced as a reinforcing agent.

Gan et al. reviewed plant-based nanocellulose composites and their properties, with a focus, especially on their thermal-related and dynamic mechanical characteristics (Gan et al., 2020). The authors pointed out that nanocellulose-reinforced composites possess outstanding properties due to the presence of nanosize filler, which makes them potential candidates to replace conventional synthetic polymer composites. It was noted that the exceptional reinforcing capability of nanocellulose is attributable to its light-weight, high stiffness, and superior mechanical strength. Nanocellulose has established to be a substantial reinforcement, even at low filler loading, where its modulus of elasticity could reach up to 150 GPa with a staggering aspect ratio up to 640, low thermal expansion coefficient (0.01 ppm·K^−1^), and high specific surface area (several 100 m^2^·g^−1^). Dominic et al. studied the effect of rice husk derived nanocellulose in replacing carbon black in natural rubber compounding (Dominic et al., 2020). The dynamic mechanical analysis demonstrated that the loss tangent (tan δ) at 60 °C is lower for the composite containing 5 wt.% of rice husk-nanocellulose and 25 wt.% carbon black compared to the composite containing 30 wt.% carbon black, implying that rice husk-nanocellulose contributes to low rolling resistance, which is a crucial parameter for green tire applications. Thus, the study has proven the potential replacement of carbon black with nanocellulose.

The maximum processing temperature of nanocellulose-based composites depends on its thermal characteristics. Nepomuceno et al. detailed that understanding the thermal behavior of nanocellulose is essential, particularly during the processing of nanocellulose and polymer composites (Nepomuceno et al., 2017). Since nanocellulose decompose at a temperature around 200–300°C, the processing temperature should be controlled at ~200°C to prevent the degradation of nanocellulose. The researchers found out that a longer duration of acid hydrolysis minimizes the thermal stability and subsequently, the degree of crystallinity of nanocellulose. Moreover, the thermal stability of nanocellulose is influenced by several factors, including the cellulose source, the processing methods used to isolate the nanocellulose and the sulfate content. Polymer matrices with the incorporation of cellulose nanocrystals usually have a low decomposition temperature in comparison to nanofibrillated cellulose owing to the existence of sulfate groups on the surface of cellulose nanocrystals because of the use of sulfuric acid during the preparation. However, the thermal stability can be enhanced by the desulfation of nanocellulose and other physical or chemical modifications (Gan et al., 2020).

Besides nanocellulose's nano-reinforcement function, it also possesses properties such as renewability, high specific surface area, biocompatibility (Zhang et al., 2017), biodegradability, optical transparency, and low thermal expansion. Nonetheless, vital properties explicitly crystallinity, surface morphology, surface chemistry, and the dimension of nanocellulose differ reliant on the source of raw material and its extraction process, which will ultimately determine their applicability (Liu et al., 2016). Yang et al. noted that nanocellulose with higher crystallinity usually possess improved mechanical and thermal properties (Yang et al., 2017). CNCs are highly crystalline stiff rod-like fragments of several hundred nanometers in length and a width or diameter around 5 to 70 nm (Liu et al., 2016; Lavoine and Bergström, 2017), typically prepared using strong acid (sulfuric acid or hydrochloric acid) hydrolysis of cellulosic fibers. Defibrillation involving acid hydrolysis dissolves the amorphous regions of cellulosic fibers, leaving tiny rod-shaped particles denoted as CNC with improved crystallinity (Moberg et al., 2017). CNCs typically possess a high specific surface area of around 150 m^2^·g^−1^ (Kaboorani and Riedl, 2015) and Young's modulus up to 170 GPa attributable to high crystallinity (typically around 50–90%) (Wei et al., 2017).

CNFs possess a width of 3–50 nm and a few micrometers of length, where the dimensions mainly depend on the conditions employed during its preparation and chemical modification (Lavoine and Bergström, 2017). CNFs possess an extended network of flexible fibers and interchangeable amorphous and crystalline regions in comparison to CNCs (Liu et al., 2016). Due to the presence of different colloidal forms, both types of nanocellulose have different physical characteristics even though they are chemically similar (Saba et al., 2017). Le Bras and co-workers characterized dielectric properties of nanocellulose from wood (cellulose nanofibrils) and algae (*Cladophora* cellulose) for electrical insulator applications (Le Bras et al., 2015). The study demonstrated a high crystallinity for *Cladophora* nanocellulose and a lower moisture adsorption capacity in comparison to CNF. Furthermore, algae nanocellulose sample was much more porous, resulting in higher dielectric loss and lower strength. It was concluded that solid-state properties of nanocelluloses might govern its dielectric properties with regards to electrical insulator applications. Table 4 depicts some of the properties and features of various forms of nanocelluloses based on the source of extraction and their preparation method.

**Table 4:** Properties and characteristics of nanocellulose substrates reliant on the cellulosic source and defibrillation method.

**Cellulosic substrate**	**Nanocellulose**	**Preparation method**	**Diameter (nm) and structural morphology**	**Average Young's modulus (GPa)**	**Apparent crystallinity (%)**	**Maximum degradation temperature (^°^C)**	**Average tensile strength (MPa)**	**Zeta potential (mV)**	**References**
Corncob residue	CNC	H_2_SO_4_ hydrolysis	5.5 ± 1.9, short rod-shaped	–	55.9	313	–	−33.8 ± 1.7	Liu et al., 2016
	CNC	Formic acid hydrolysis	6.5 ± 2.0, long rod-shaped	–	63.8	360	–	−14.3 ± 0.4	
	CNF	TEMPO-mediated oxidation	2.1 ± 1.1, twisted structure	–	49.9	305	–	−23.1 ± 2.3	
	CNF	PFI refining	43.1 ± 25.3, twisted	–	52.1	336	–	−40.3 ± 1.5	
Stalks of wheat straw (*Triticum paleas*)	CNF	H_2_SO_4_ hydrolysis and ultrasound treatment	10–40, a mesh-like multilayer structure	11.45	72.5	ca. 400	42.3	–	Barbash et al., 2017
Cornhusk	CNC	H_2_SO_4_ hydrolysis	26.9 ± 3.35, short rod-shaped	–	83.5	351	–	−34.6 ± 2.3	Yang et al., 2017
	CNF	TEMPO-mediated oxidation	10.48 ± 1.83, slender interconnected webs	–	72.3	279	–	−69.4 ± 1.7	
	CNF	High-intensity ultrasonication	20.14 ± 4.32, slender interconnected webs	–	53.4	348	–	−24.3 ± 2.5	
Banana pseudostem	CNF	High-pressure homogenization	30–50, entangled network of polydisperse bundles	–	67.0	337	–	–	Velásquez-Cock et al., 2016
Cotton	CNC	H_3_PO_4_ hydrolysis	31 ± 14, rod-like shape	–	81.0	325	–	–	Camarero Espinosa et al., 2013
Ushar (*Calotropis procera*) seed fiber	CNC	H_2_SO_4_ hydrolysis	14–24, needle shape	–	70.0	ca. 330	–	–	Oun and Rhim, 2016
	CNF	TEMPO- oxidation	10–20, web-like shape	–	59.0	316	–	–	
Bacterial strain *Komagataeibacter xylinus* (BCC529)	BNC	Static culture for 96 h at 30 °C	29.13 ± 6.53, denser network structure	0.72	47.4	335	0.235	−44.1 ± 0.9	Gao et al., 2020
	BNC	Agitated culture: 300 rpm at 30 °C	29.51 ± 8.03, loose and porous network	–	22.1	310	–	−46.5 ± 1.5	
Kenaf (*Hibiscus cannabinus L.)* fiber	CNC	H_2_SO_4_ hydrolysis and ultrasonic treatment	10–28, morphology not defined	−	80.0	ca. 420	61.4	−	Barbash and Yashchenko, 2020

*TEMPO, 2,2,6,6-tetramethylpiperidine-1-oxyl*.

### Surface Modification of Nanocellullose

The study of functional nanoscale materials has emerged as an attractive field of research since they possess vastly improved properties and characteristics, enabling these high value-added substrates to be applied in the field of materials science. Cellulose is a natural polysaccharide, and an abundant biopolymer serves as building blocks in the structural hierarchy (Lin et al., 2012). Concerning the vastly hydrophilic nature of nanocellulose owing to the existence of OH groups on their surface, the surface chemistry can be tuned chemically, physical interactions (Huang et al., 2020), and biological approaches. Surface functionalization can be carried out during the preparation step or post-production of nanocellulose (Wei et al., 2017). These modifications lead to attaining desirable properties, which, in turn, enhance their effectiveness for a given application (Lin et al., 2012; Afrin and Karim, 2017; Liang et al., 2020; Tao et al., 2020). Through the incorporation of any chemical functionality, the surface of a nanocellulosic material can be modified the way it reacts with foreign substances (George and Sabapathi, 2015). Lin et al. remarked that polymeric matrices with improved reinforcement, i.e., enhanced thermal and mechanical performances, can be obtained through the surface modification of polysaccharide nanocrystals (Lin et al., 2012).

On the contrary, cellulose nanocrystals not only consist of primary reactive sites (i.e., hydroxyl groups), they possess high surface area to volume ratio, making CNC highly reactive and easy to be functionalized. Cellulose nanocrystals are chemically modified in order to impart stable positive or negative electrostatic charges on the surface for a better distribution of particles and to enhance their compatibility (Kaboorani and Riedl, 2015). In 2019, Lu and co-workers studied the interfacial compatibility of hydroxyapatite modified nanocellulose with polylactic acid (PLA) matrix to overcome PLA's inherent hydrophobicity (Lu et al., 2019). Morphological study *via* transmission electron microscopy, Fourier transform infrared spectroscopy, and X-ray diffraction analysis corroborated successful structural modification of nanocellulose obtained from cotton pulp. The study revealed that hydroxyapatite modified nanocellulose enhanced the mechanical properties of PLA based nanocomposite films pertaining to the occurrence of strong hydrogen bonding interaction at the interface, which resulted in a good dispersion in the PLA composite. Moreover, the surface modification significantly improved the tensile strength, tensile modulus, and thermal stability of the nanocomposite, signifying that hydroxyapatite modified nanocellulose is a good reinforcing material for PLA.

According to previous literature (George and Sabapathi, 2015; Afrin and Karim, 2017; Daud and Lee, 2017; Huang et al., 2020), the surface of cellulose nanocrystals can be chemically modified using numerous methods, mainly covalent surface modification including sulfonation, polymer grafting, oxidation, esterification, nucleophilic substitution, etherification, silylation, and carbamation. In a recent study, polyacrylamide has been grafted onto cellulose nanocrystals (CNC) to integrate into poly(vinyl alcohol) (PVA) employing a solution casting method to reinforce nanocomposite films. Infrared spectroscopy affirmed the occurrence of strong hydrogen bonding on the surface of CNC, i.e., between hydroxyl groups of PVA matrix and polyacrylamide chains, which improved the interfacial compatibility. The study revealed that prepared nanocomposite films at 0 and 50% relative humidity achieved an increase in elastic modulus. The thermogravimetric analysis demonstrated the enhanced thermal stability of reinforced PVA-nanocomposites, corroborating the significance of surface modification of CNC through grafting in view of enhancing its thermal and tensile properties (Li B. et al., 2020). In a study carried out by Tang et al. discovered that hydrophobically modified cellulose nanofibrils through the grafting of cinnamoyl chloride and butyryl chloride displayed favorable surface properties, capable of stabilizing oil-water emulsions (Tang C. et al., 2019). They noted that nanocelluloses possessing high surface charge density do not effectively stabilize Pickering emulsions, which limit their application as interfacial stabilizers. Thus, surface modification *via* grafting hydrophobic polymers onto nanocelluloses improve their wettability by the oil phase, resulting in reduced interfacial tension.

Moreover, the use of adsorbing surfactants (Kaboorani and Riedl, 2015) and polymer coatings (Islam et al., 2013) have also been employed.

Bertsch and Fischer discussed on the adsorption and interfacial structure of nanocelluloses (NC) at the fluid interface, where nanocelluloses with their native hydrophilic and hydrophobized surfaces impart essentially different interfacial structure and adsorption characteristics (Bertsch and Fischer, 2019). It was noted that nanocelluloses are green alternative for the stabilization of fluid interfaces. The adsorption of NCs at oil-water interfaces facilitates the formation of stable and biocompatible Pickering emulsions. Furthermore, the review study elaborated that unmodified NCs cannot stabilize foams. In contrast, NCs with covalent surface modifications or through the adsorption of surfactants could hydrophobize its surface (contact angle, θ > 90Â°), consequently stabilize foams or inverse and multiple emulsions. Many pioneering applications already employ nanocellulose-stabilized colloids, for instance, preparation of 3D-printing inks (Huan et al., 2018, 2019), novel bio-nanocomposites (Reid et al., 2019; Bielejewska and Hertmanowski, 2020), and in gastric stable delivery systems (Bai et al., 2019; Liu and Kong, 2019), pertaining to NCs' outstanding stability and biocompatible nature. Xiang et al. discovered that cellulose nanofibrils form more stable foams compared to cellulose nanocrystals, attributed to cellulose nanofibrils ability to spread into the bulk, ensuing enhanced interfacial and bulk elasticity (Xiang et al., 2019). Bai et al. investigated the stabilization of concentrated edible oil-in-water Pickering emulsions by modifying the surface of naturally derived cellulose nanocrystals with a food-grade cationic surfactant (ethyl lauroyl arginate) (Bai et al., 2018). The researchers revealed that as surfactant-covered NCs are more hydrophobic, their surfaces impart lower surface tension and higher surface coverage, contributing to enhanced electrostatic stabilization and emulsifying ability.

Some of the physical surface functionalization techniques may include electric discharge (plasma treatment), ultrasonic treatment, irradiation, and surface fibrillation (Islam et al., 2013). Enzymes can also be used to modify the surface of nanocellulose, for instance, Afrin and Karim (2017) employed two approaches in their study; (a) direct modification, where the enzyme directly gets in contact with nanocellulose, and (b) indirect enzyme-mediated modification. They concluded that the enzymatic approach to produce nanocellulose and to further functionalize them is a viable greener modification method compared to chemical modification route. Islam et al. (2013) pointed out that the main obstacle in modifying cellulose nanofibrils (CNF) is that the approach needs to alter only the surface without disrupting the morphology to preserve the crystal structure. According to Robles et al. (2015), surface modification of CNC and CNF with silanes generates hydrophobic surfaces, which increase their stability.

**Figure 5:**
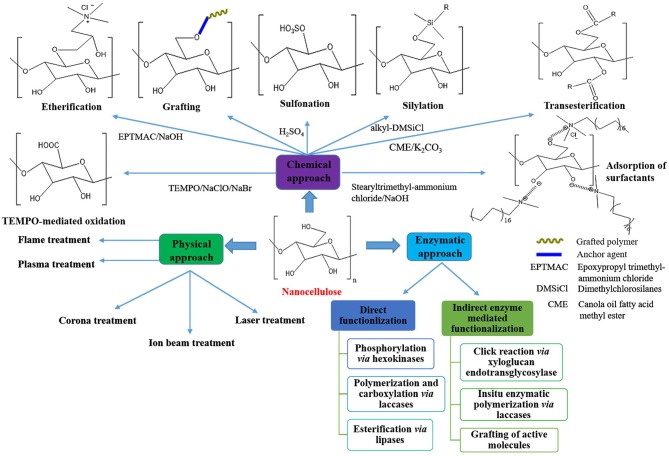
Schematic representation of the most commonly used surface modification routes of nanocellulose.

Hydrolases and oxidoreductases are two of the most commonly used classes of enzymes. Glycosidases, proteases, and lipases are the frequently used hydrolase enzymes, while in the case of oxidoreductases, laccase, tyrosinase, and peroxidase are the majorly employed enzymes. Concerning biomedical applications, nanocellulosic materials functionalized via enzymatic approach possess a significant advantage over the chemical route, which avoids the toxicity of the modified product (Karim et al., 2017). Besides, based on the presence of a high density of hydroxyl groups, BNCs can also be modified (Wang et al., 2020). Kalhori and Bagherpour (2017) pointed out that the utilization of bacterial cellulose for engineering applications is a growing topic of interest among materials scientists and engineers. For instance, Akhlaghi et al. (2020) investigated the susceptibility of bacterial nanocellulose fibers as reinforcement in cement composites, in which they observed that BNCs improved the mechanical properties of cement mortars. Consequently, the development of modified nanocellulose substrates with intact surface properties through “sustainability” is still an uphill task in nanocellulose research. Figure 5 depicts a schematic diagram representing the most commonly used surface modification routes of nanocellulose, while Table 5 portrays some previous studies on surface modification of nanocellulose substrates along with their salient features and applications.

**Table 5:** Previous studies on surface modification of nanocellulose substrates along with their salient features and applications.

**Cellulosic source**	**Nanocellulose**	**Preparation method**	**Surface modification strategy**	**Salient features**	**Application**	**References**
Eucalyptus dry lap wood pulp	CNC	H_2_SO_4_ hydrolysis	Transesterification with canola oil fatty acid methyl ester	Higher hydrophobicity and thermal stability	Hydrophobic coatings and reinforcing agents to hydrophobic polymer for nanocomposites	Wei et al., 2017
Blue agave (*A. tequilana*) bagasse	CNF	High-pressure homogenization	Silanization with 3-aminopropyl triethoxysilane	Enhanced mechanical properties and hydrophobicity	Additive in poly(lactic acid) to form strengthened composites	Robles et al., 2015
	CNC	H_2_SO_4_ hydrolysis	Esterification with dodecanoyl chloride			
Softwood pulp	CNF	TEMPO-mediated oxidation	Grafting of cetyltrimethylammonium bromide surfactant	Increased hydrophobicity and thermal stability	Improve the redispersibility of TEMPO-oxidized CNFs in N, N-dimethylformamide	Qu et al., 2019
Softwood sulphite fibers	CNF	Successive grinding	Sonication in the presence of lactic acid	Rapid water draining and enhanced mechanical properties	Performance-enhancement additive in traditional papermaking	Sethi et al., 2018
Sugarcane bagasse	CNC	H_2_SO_4_ hydrolysis	Functionalization using adipic acid	Improved dispersion and thermodynamic wetting	Reinforcements for hydrophobic polymer matrices	Ferreira et al., 2018
Bacterial strain *Gluconobacter xylinus* (53582)	BNC	Static culture at 26°C for 168 h	Incorporation of polyvinyl alcohol and Ag nanoparticles	Outstanding antimicrobial and mechanical properties	Packaging films for the food industry	Wang et al., 2020
Sea pineapple (*Halocynthia roretzi*)	CNF	TEMPO-mediated oxidation	Grafting of polyethylenimine	Well-developed pore structure with excellent adsorption ability	To develop circular routes in recovering metals and reuse them directly	Hong et al., 2020
Aspen kraft pulp	CNC	H_2_SO_4_ hydrolysis	Oxidizing CNC by sodium periodate followed by covalent immobilization of black wattle tannin	Better regeneration and reusability with high metal adsorption capacity	Novel nanocomposite to eliminate contaminants from industrial effluents	Xu et al., 2017

## Nanocellulose Based Nanocomposites

The unique and attractive characteristics of cellulose nanocrystals are already well-documented, which pushed the scientific community to focus on the development of practical applications for this nanoscale material (Kiziltas et al., 2013; Pandey et al., 2015; Thakur, 2015b; Abitbol et al., 2016; Jawaid et al., 2017; Wang X. et al., 2017; Nascimento et al., 2018; Salimi et al., 2019; Younas et al., 2019). The employment of CNCs as reinforcing agent of polymers is one of the most studied area in composites field. A nanocomposite is considered as a heterogeneous mixture, which contains two or more different components with substantially various physicochemical features. By definition, such mixture consists of a homogeneous matrix (polymer or biopolymer) constituent that is reinforced by a stiffer, stronger component with a small amount of nanosized of organic or mineral fillers of specific shape, size, and surface chemistry (Abdul Khalil et al., 2019). CNCs have been revealed to be an interesting nanofiller owing to their chemical structure (abundance of -OH groups), reactivity, high specific surface area, mechanical, thermal and optical properties, even when incorporated at low concentrations (Chen et al., 2019). Broadly, it is pointed out that the incorporation of CNCs into a polymeric matrix enhances the tensile strength and decreases the elasticity. Such behavior can be assigned to the strong intermolecular linkages such as covalent bonds, van der Waals forces, mechanical interlocking and molecular entanglement between CNCs and the polymeric matrix (Pires et al., 2019). Besides that, compared to the conventional nanofillers such as carbon black, mica, silica, nanoclay, and non-aluminum oxide, CNCs present lower health and environmental negative impacts (Ng et al., 2017). Various processing methods have been developed to produce CNC-based nanocomposites. The most important ones are the solution casting, melt extrusion, ball milling, injection molding, compression molding, precipitation routes, 3D printing, layer-by-layer assembly, wet- and elector-spinning, and micropattering techniques (Oksman et al., 2016; Dufresne, 2018; Nascimento et al., 2018; Thomas et al., 2018; Sharma et al., 2019). This kind of nanocomposites can be used in several scientific area and industries such as packaging, automotive, aerospace, paints and coatings, adhesives, hydrogels, nanobarriers, inks and printing, fire retardants, cementitious materials, and defense, to name a few. CNC-based composites presenting wide range of applications have been comprehensively reviewed in recent years (Malucelli et al., 2017; Ilyas et al., 2018; Klemm et al., 2018; Thomas et al., 2018; Dufresne, 2019; Fiss et al., 2019; Naz et al., 2019; Sharma et al., 2019; Montes et al., 2020).

Nonetheless, most synthetic polymers are hydrophobic materials, giving rise to week adhesion, reduced dispersion, and poor wettability of CNCs with the polymer matrix. Furthermore, the use of non-polar media causes a poor dispersion of CNCs due to their ability to generate aggregates owing to the presence of polar chemical groups and the high surface energy of these nanoparticles (Dufresne, 2019; Younas et al., 2019). Hence, surface modifications *via* covalent binding, surfactants, ionic interactions, reductive amination, physical adsorption, and molecule/polymer grafting have been demonstrated as efficient approaches for enhancing the compatibility between nanocomposite components, thereby improving the dispersion and interaction between them (Thakur et al., 2013; Abitbol et al., 2016; Younas et al., 2019; Nigmatullin et al., 2020). Moreover, the processing temperature of CNCs and some engineering plastics such as polyethylene and polypropylene is another shortcoming, which requires the development of high-performance natural fiber-reinforced composites (Gopi et al., 2019).

CNCs have been employed to reinforce a wide range of polymer matrixes. Both thermoplastic polymers and thermosets have been reinforced with CNCs to produce high-quality and cost-effective materials (Lu et al., 2014). Many in-depth research papers and reviews dealing with CNC-based thermoplastics have been published over the past 20 years, covering the preparation methods, potential applications, shortcomings and advantages. Various polymers such as polymethyl methacrylate, polybutyl methacrylate, polyvinyl chloride, poly exo-ethylene, ethylene oxide-epichlorohydrin co-polymers, polyurethane, polycarbonate, poly lactic acid, poly vinyl acetate, and polyvinyl alcohol (Abitbol et al., 2016; Nandi and Guha, 2018). The mechanical features of this kind of nanocomposites are deeply affected by the interfacial adhesion between CNCs and the polymer matrix, which can enhanced by caring out specific modifications on these components (Gopi et al., 2019). On the other hand, the combination of CNCs and thermosets (unsaturated polyesters, some polyurethanes, epoxy and phenolic resins) in nanocomposites aims to couple attractive features from each compound in a synergetic manner (Peng et al., 2017; Yue et al., 2018). CNCs provide better strength and stiffness along with resistance to corrosion. These nanfillers could be beneficial in reducing the internal stresses introduced during curing process and can increase the cross-linking density and impart nanocomposites with significantly improved toughness (Miao and Hamad, 2019). Recently, Yue et al. have pointed out that the use of modified CNCs enables physical interlocking points in the cured epoxy matrix restricting chain mobility, and the homogeneity of the dispersion is a key factor, which enhances the filler-matrix dispersion contributing to the significant improvement in storage modulus and glass transition (Yue et al., 2018). Furthermore, the formation of CNC-polymer matrix network depends on the percolation threshold that relies on the aspect ratio of the nanofillers and strength of the filler/filler interactions. Nevertheless, despite such nanocomposites present interesting characteristics compared to thermoplastics (Gopi et al., 2019), they show some drawbacks such as the high curing temperature and time as well as some recycling issues (Liu et al., 2012). Notwithstanding, with constancy proposing environmental and sustainable concerns in the last decades, the employment of natural fillers and polymer matrixes from natural and renewable resources in nanocomposites have drawn more and more attention. Various bionanocomposites have been produced using CNCs as nanofillers and natural polymer matrixes such as chitosan, gelatin, proteins, cyclodextrin, starch, gluten, alginate, natural rubber, xanthine, and cellulose derivatives (carboxymethyl cellulose, hydroxypropylcellulose, regenerated cellulose and cellulose diacetate) (Younas et al., 2019). This latter class may find potential real applications in the near future. Recently, despite much progress has been made, more effective and efficient methodologies and strategies require to be developed to obtain nanocomposites with optimal features, encompassing the scale up to industrial level at economic way (Vilarinho et al., 2018). To resolve this, efforts are being made to seek for new approaches capable of improving the existing processes or promoting large scale synthesis.

**Figure 6:**
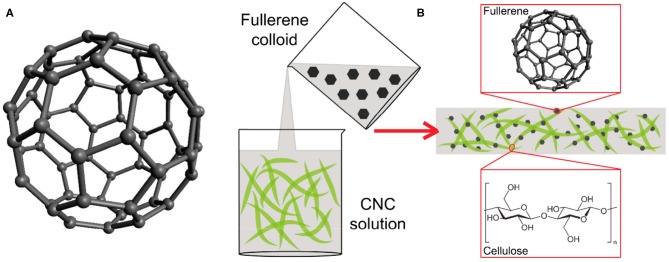
Scheme of fullerene C_60_ **(A)** and of the preparation and structure of nanocellulose/fullerene composites **(B)**. Reprinted from Bacakova et al. (2020) as distributed by creative common license CC BY license, MDPI publisher.

On the other hand, owing to the surface characteristics of CNCs, they can be modified by oxidation of hydroxyl groups using the TEMPO-oxidation or ammonium persulfate method to produce multifunctional hybrid nanomaterials coupled with metal or metal oxide nanoparticles such as Ag, ZnO, CuO, and Fe_2_O_3_, as recently described by Oun et al. (2020). These composites have a high potential to be employed in different fields encompassing food packaging and other biotechnological applications. Nanocomposites, containing nanocellulose and nanocarbons such as graphene, graphene oxide, carbon nanotube, nanodiamond, and graphene, are considered as newly emerging smart hybrid materials, where nanocellulose component acts as a dispersing agent (Bacakova et al., 2020; Miyashiro et al., 2020). For instance, CNCs were considered ideal for immobilization of fullerene nanoparticles. A scheme of preparation of CNC/fullerene composite is depicted in Figure 6. Such composite showed a higher radical scavenging capacity *in vitro* than fullerenes alone, and therefore are promising for biomedical application in antioxidant therapies, e.g., as components of skin care product (Awan et al., 2016). Nanocellulose/nanocarbon composites present outstanding properties such as stretchability, flexibility, high mechanical strength, tunable optical transparency, tunable electrical and thermal conductivity, photothermal and photodynamic activity, nanoporous character, and high adsorption capacity. Consequently, they are prominent for a wide range applications such as food packaging, water purification, energy harvesting, storage and conversion, shape memory devices, construction of fire retardants, and biomedical uses (Bacakova et al., 2020). Other CNC hybrids based on metals, oxides, mineral salts, and non-metallic elements have been also investigated. These nanocomposites exhibited numereous innovative features due to synergetic effects, which are unachievable by nano-structured materials alone (Zhang et al., 2020). For instance, CNC-loaded with noble metal nanoparticles found applications in the field of antibacterial, biomedical, protein/enzyme immobilization, catalysis, photoelectric materials, and sensing. However, some challenges in controlling size, shape, distribution uniformity, and density of nanoparticles on CNCs need to be overcome. Moreover, the interactions and the complex mechanisms between these nanomaterials from a fundamental point of view have to be illucidated. Besides, more environmentally friendly and green synthetic approaches should also be considered to meet the sustainable development and the strong demands. Nonetheless, the fabrication and exploring CNC loaded with nano objects and their extended uses will remain as one of the key focuses of future research activities.

## Nanocellulose for Biomedical Applications

The advances in the area of nanomaterials, with outstanding features and various structures, have attracted more interest for their use in biomedical applications. The coupling of multidisciplinary fields such as life science, biology, physics, chemistry, and engineering has long assisted the evolution of nanobiomaterials, which can be rationally designed from biological or synthetic materials, for biomedical utilizations (Kim D. et al., 2019; Pires et al., 2019). Since the pioneer work of Kramet et al. appeared in June 2006 giving light to the potential employment of nanocellulose as biomaterial for constructing tissues replacements (Kramer et al., 2006), extensive research activities have been conducted and others continue to appear worldwide focusing on the employment of nanocellulose such as CNCs in several fields of medicine encompassing tissue regeneration, tissue repair, substitute implants, biosensing, drug delivery, hemodialysis membranes, absorbable hemostats, biocatalysts, anti-bacterial etc. (Lin and Dufresne, 2014; Trache, 2018; Bacakova et al., 2019; Karimian et al., 2019; Moohan et al., 2020). Some important applications will be discussed below, whereas detailed discussions dealing with other medical uses of CNC-based materials can be found in other reviews (Golmohammadi et al., 2017; Grishkewich et al., 2017; Reiniati et al., 2017; Phanthong et al., 2018; Seabra et al., 2018; Wohlhauser et al., 2018; Bacakova et al., 2019; Carvalho et al., 2019; Du et al., 2019; Dufresne, 2019; Jin et al., 2019; Kim D. et al., 2019; Luo et al., 2019; Naz et al., 2019; Pires et al., 2019; Salimi et al., 2019; Sharma and Bhardwaj, 2019; Sharma et al., 2019; Shojaeiarani et al., 2019; Tan et al., 2019; Younas et al., 2019; Mokhena and John, 2020; Moohan et al., 2020).

CNCs have shown great promise owing to their biodegradability, biocompatibility, high surface area-to-volume ratio, interesting thermal, optical, electrical, barrier and mechanical characteristics, no/low toxicity, self-assembly behavior, crystallinity, rheology, potential versatility in terms of functionalization and modification (Plackett et al., 2014; Jorfi and Foster, 2015; Karimian et al., 2019). It has been reported that CNCs are non-cytotoxic, non-immunogenic and do not contribute to serious environmental hazards, and suggested as a tissue culture medium to assist cell proliferation (Ilyas et al., 2018). Nevertheless, recent research activities have revealed that CNCs may cause inflammatory response, mages, induce oxidative stress and are able to enter cells, what is dominated by the nanometric size as well as the nature of surface chemistry, and thus CNC-based materials may influence the toxicity in different manners (Thomas et al., 2018). However, such immunogenicity and cytotoxicity can be modulated by CNC physicochemical features through endowing an electrical charges or by the functionalization of specific chemical groups (Bacakova et al., 2019). Moreover, further investigations on the CNC toxicity are required to have a better insight for the next applications in the biomedical field (Seabra et al., 2018).

Efficient drug delivery systems exhibit important features such as targeting, improved solubility, controlled drug release, reduced clearance, drug stability, and therapeutic effect. Some endeavors have been devoted to utilize CNCs as an appropriate pharmaceutical excipient and carrier owing to their colloidal stability, high surface-to-volume ratio and the negative surface charge, which allowed loading charged/neutral drugs, controlling the release of active compounds, and transporting the genes to the target cells (George and Sabapathi, 2015; Grishkewich et al., 2017; Tan et al., 2019). However, the hydrophilic character and the low drug-loading behavior of CNCs limit it use in pristine form. Therefore, to improve binding of hydrophobic drugs, a wide range of surface modifications of CNCs have been carried out based on the chemical introduction of chemical groups owing to the presence of reactive functional groups on CNC backbone (Lin and Dufresne, 2014; Plackett et al., 2014; Salimi et al., 2019). Nevertheless, maintaining the morphology of CNC crystals after modification processes remain an additional challenge that necessitates further improvements to enhance the efficiency of CNC-based drug delivery systems (Karimian et al., 2019). For instance, Wan et al. developed a new approach to prepare hyperbranched polymers-functionalized CNCs through direct anionic polymerization utilizing glycidol as the monomer and surface hydroxyl groups of CNCs as initiator. The peripheral end functional groups of the modified CNCs were then converted to hydrazide groups, which could be used for loading anticancer drugs, such as epirubicin, through the formation of hydrazone bonds with pH-responsiveness (Wan et al., 2019). The authors suggested that epirubicin could be released from CNCs-based carriers with pH-responsive behavior and that the obtained drug-containing complexes could preserve their anticancer capability. Recently, Tang et al. prepared a novel colon-targeted drug release system by conjugating of maleic anhydride CNCs (MCNC) with model drug (tosufloxacintosilate) (Tang et al., 2018). They revealed that the model drug could be entrapped effectively by MCNC, and hence excellent behavior for colon-targeted release is found. In another work, Ntoutoume et al. developed complexes containing CNC/curcumin/cyclodextrin to target colon and prostate cancer cells (Ntoutoume et al., 2016). They demonstrated that such complexes present an effective antiproliferative effect on cancer cells compared to curcumin alone. On the other hand, CNC-based hydrogels present interesting bioavailability and can provide superior drug delivery capacity due to their high surface area and open pore structure. Intense research works have been recently performed as demonstrated by the recent published reviews (Du et al., 2019; Shojaeiarani et al., 2019). More recently, Xu et al. fabricated a novel nanocomposite hydrogel using CNCs and chitosan and employed it as a carrier for the controlled delivery of theophylline (Xu Q. et al., 2019). They reported that such biocomposite exhibited excellent drug-controlled release behavior and can be employed as prominent carrier for gastric-specific drug delivery. In another research work, Jeddi et al. prepared carbocymethyl CNC, which is utilized in bilayer alginate-chitosan hydrogel beads formulation to produce smart, friendly, and magnetic sensitive hydrogels beads and successfully applied as a carrier for dexamethanose delivery (Jeddi and Mahkam, 2019). They claimed that the developed simple green manufacturing method using economical feedstocks provided a highly prominent carrier for drug delivery.

Tissue engineering (TE), a fast growing area of biomedical science, has emerged as a prominent approach to develop biological substitutes for repairing, treatment or regenerating lost or damaged tissue or organ based on the application of principles and methodologies of engineering, chemistry, biological sciences, and medicine (Du et al., 2019; Shojaeiarani et al., 2019). CNC-based materials have received a tremendous attention and are actively investigated through TE approach since they present all the requirements of TE technology such as sustainability, biodegradability, biocompatibility, water retention, water absorption, better mechanical features, enhance cell adhesion, growing, and differentiation (Abitbol et al., 2016; Mokhena and John, 2020). Various manufacturing techniques have been developed for TE scaffolds such as electorspinning, freeze-drying, crosslinking, solvent casting, and 3D printing (Kim D. et al., 2019; Luo et al., 2019; Moohan et al., 2020). CNCs have constantly shown to be promising component in several formulations for TE applications such as in the repair of ophthalmic, hepatic, muscular, neural, vascular, skin, cartilage, cardiac, and bone tissue, especially after their chemical and physical characteristics have been modified. CNCs can reinforce many polymeric matrices and are compatible with various biological materials such as poly(lactic acid), chitosan, silk fibronin, alginate, collagen, apatite, and gelatin (Grishkewich et al., 2017; Thomas et al., 2018; Shojaeiarani et al., 2019). Many formulations have been developed for widespread application and prominent results have been achieved in the recent years (Gopi et al., 2019; Kim D. et al., 2019; Naz et al., 2019; Moohan et al., 2020). For instance, Shaheen et al. fabricated chitosan/alginate/hydroxyapatite/CNC scaffold using freeze-drying method for bone tissue engineering (Shaheen et al., 2019). They confirmed that the incorporation of CNCs improved the mechanical as well as physical properties of scaffolds, and cell adherence and proliferation were enhanced. In another work, Osorio et al. prepared hydrazone cross-lined CNC aerogels as viable bone tissue scaffolds (Osorio et al., 2019). They claimed that the obtained aerogels are flexible, porous, and efficiently facilitate bone growth after they implantation if bone defects. More details of other interesting applications of CNCs for other TE can be found in some recent reviews (Bacakova et al., 2019; Gopi et al., 2019; Kim D. et al., 2019; Luo et al., 2019; Naz et al., 2019; Pires et al., 2019; Sharma et al., 2019; Mokhena and John, 2020; Moohan et al., 2020).

Human skin, the interface between the body and the environment, plays a prominent role as protective layer and physicochemical barrier against aggressions. Skin injuries, which happen in daily life, necessitate an efficient treatment and proper management to avoid severe illnesses or even mortality (Du et al., 2019). Wound dressings is a practical and efficient way to heal skin injuries and protect the area from the risk of infection from microorganisms. An effective wound dressing material needs to satisfy a number of features, i.e., it should be non-allergic and non-toxic, induces wound healing, eliminates dead spaces and non-viable tissues and control odors, avoids further inflammation, cleans the injured tissue, minimizes/eliminates pain, controls and prevents microbial biofilms, absorbs excess exudate and toxins, has good permeability to oxygen, maintains suitable moisture at the surface, and must be simple to eliminate without any trauma to the wound (Mogoşanu and Grumezescu, 2014; Hamedi et al., 2018). The application of CNCs to wound healing was recently reported is many investigations, and promising achievements have been reached owing to their useful properties as well as their ability to be functionalized (Bacakova et al., 2019; Dufresne, 2019; Kim D. et al., 2019; Miao and Hamad, 2019; Naz et al., 2019; Sharma et al., 2019; Shojaeiarani et al., 2019; Mokhena and John, 2020; Moohan et al., 2020). For example, Yin et al. synthesized hydrogels based on gelatin (GA), hyaluronic acid (HA) and CNCs by crosslinking and freeze-drying (Yin et al., 2019). They obtained hydrogels through the formation of amide bond and hydrogen bonding between hydrogel components. The authors claimed that the introduction of CNCs improved the characteristics of hydrogels and played a prominent role according to the swelling and rheology behavior. The cell culture exhibited that NIH-3H3 cells can attached to, grow, and proliferate well on the GA-HA-CNC hydrogels, confirming their potential application in wound dressing field. Recently, Dehkordi et al. prepared a novel CNC-reinforced hyaluronic acid composite containing nanochitosan loaded with granulocyte macrophage colony stimulating factor (GM-CSF) as an efficient candidate for wound healing (Dehkordi et al., 2019). The resulted composite exhibited interesting features such as high swelling capacity, suitable mechanical characteristics and controlled release of GM-CSF. The authors reveled that this composite enhanced granulation formation, improved re-epithelialization, and decreased inflammatory reaction, suggesting that such composite can be possibly used in clinical practice for wound treatment. Most recently, Shojaeiarani et al. reported that thermo-responsive injectable hydrogels reinforced by cellulose nanocrystals have the capability to efficiently orient medicine to narrow or deep-opening wounds and the aptitude of continued release of antibiotics, which are appropriate in wound healing uses (Shojaeiarani et al., 2019). Some other specialized reviews have been recently published on this topic (Alavi, 2019; Du et al., 2019; Dufresne, 2019; Kim D. et al., 2019; Shojaeiarani et al., 2019; Mokhena and John, 2020; Moohan et al., 2020).

Biocatalysts, as biological substances, can be used for the initiation, modification, and promotion of the chemical reaction rates. They present numerous advantageous compared to the conventional chemo-catalysts for biomedical and healthcare applications since they are biocompatible and selective, exhibit higher catalytic activity, increased susceptibility, improved enzyme-substrate affinities, and reusability, and may be prepared via green chemical processes under mild conditions (Karimian et al., 2019; Lin N. et al., 2019). In recent years, CNC has been widely used as a novel matrice for the immobilization of enzymes/proteins (Grishkewich et al., 2017; Karimian et al., 2019). It is revealed that enzyme/CNC can considerably enhance the catalytic activity, enantioselectivity, and stability of the enzymes (Sunasee et al., 2016). An effective chemoenzymatic approach for immobilizing proteins onto CNC scaffolds has been reported by Uth et al. An oligo-GLy sequence was conjugated to CNC surface, which could be recognized and cleaved by sortase A to allow for protein immobilization, following the conversion of the surface hydroxyl to aldehyde groups (Uth et al., 2014). The advantageous of this method is that the protein grafting can be site specific and region-specific at physiological conditions, allowing the protein to maintain its structure without affecting its activity (Grishkewich et al., 2017). Thus, taking into account of the benefit of the high dispersible CNC scaffold, such approach can be applied for several proteins and bioactive molecules. In another work, Cao et al. have produced magnetic CNC as an enzyme support for immobilization of Pseudomanas cepacialipase (PCL) (Cao et al., 2016). The authors demonstrated that the use of CNC enhances the stability and solvent tolerance owing to the increase of the enzyme structure rigidity. This biocatalyst seems to be able to effectively catalyze the hydrolysis of ketoprofenethyl ester with high yield. Recently, Huang et al., prepared a novel nanobiocatalyst by immobilizing penicillin acylase onto magnetic CNCs and assessed its use for the efficient synthesis of cefaclor (Huang et al., 2018). The authors revealed that the obtained nanobiocatalyst exhibited significantly enhanced stability and manifested higher enzyme-substrate affinity and catalytic efficiency with higher yield. Such biocatalyst can be considered as a prominent and effective substance for biocataltic reactions, which may be used to produce semi-synthetic antibiotics. More recently, Wu et al. have improved the biocatalysis of cefaclor through a new approach based on the synthesis of penicillin acylase immobilized on magnetic CNCs in deep eutectic solvents (Wu et al., 2019). This approach allowed obtaining a yield of 91% of cefaclor.

Despite interesting positive results and achievements on the application of CNC-based materials for biomedical uses have been reached, further investigations on the long-term biocompatibility and toxicology should be carried out in addition to the implementation of the validation of these CNC-based biomaterials using the standards and methodologies applied by the competent authorities.

## Nanocellulose as Pickering Emulsifiers

Pickering emulsions have drawn more interest in recent two decades owing to new developments and insights in material science and engineering (Fujisawa et al., 2017). Pickering emulsion refers to solid-stabilized emulsions, where solid particles are introduced to well stabilize emulsions, instead of traditional surfactants, through the adsorption process on the surface of emulsion droplets and the lowering of the interfacial tension to generate a protective coating that obstructs the flocculation and coalescence of droplets owing to the instruction of steric or electrostatic repulsive forces (Tang J. et al., 2019). Among several solid stabilizes, it was recently exhibited that CNCs can be used as Pickering emulsion stabilizers owing to their amphiphilicity, unique nanosizes and promising features such as renewability, biocompatibility, biodegradability, and chemical stability. One of the pioneers research group in the field is that of Capron, who demonstrated in 2011 that unmodified CNCs can efficiently adsorb oil-water interfaces and generate deformable and highly stable oil-in-water emulsions, showing the amphiphilic character of CNCs, demonstrated by molecular organization at crystalline surfaces, with the exhibition of affinity between both hydrophobic and aqueous phases (Capron, 2018). Despite the known hydrophilic character of cellulose (abundance of –OH groups), a more hydrophobic edge plane constituted by only CH groups has been recognized as (200) crystalline plane for Iβ allomorph, and (110) Iα allomorph. This hydrophobic edge plane is appeared responsible for wetting CNCs at the oil/water (O/W) interface, and hence its accessibility to oil droplets governs the establishment of thermodynamically stable oil droplets (Tarimala and Dai, 2004; Goi et al., 2019). Typically, unmodified CNCs are revealed to generate only oil-in-water emulsions. Besides that, it is reported that the nature of CNCs source, the pretreatment of biomass and the isolation method do not significantly influence the ability to form the Pickering emulsions, because native cellulose has common crystal allomorphs. However, the morphology, shape, aspect ratio, specific surface as well as the quantity of CNCs involved may cover in different ways the droplets and modify the behavior of the emulsion (Fujisawa et al., 2017; Capron, 2018). Aside from their biocompatibility and abundance, another benefit of utilizing CNCs is that they can be easily modified. Various modification methodologies, encompassing covalent bonding through the insertion of functional chemical groups or surface active species (surfactants, polymers, or proteins) and non-covalent bonding through physical interactions such as electrostatic interaction, hydrogen bonding, and van der Waals force, have been developed to tailor their characteristics and enhance the emulsion stability (Hu et al., 2015b; Bai et al., 2018; Liu et al., 2018; Pindáková et al., 2019; Tang J. et al., 2019). The CNCs functionalization controls the type of emulsion. Thus, the hydrophobic character of the CNC modification produces water-in-oil (W/O) emulsions with high stability. The combination of the (O/W) and (O/W) emulsions produces double emulsions (W/O/W or O/W/O) via coupling of unmodified and modified CNC (Kalashnikova et al., 2013; Hu et al., 2015a).

CNC-based Pickering emulsions find applications in various fields such as food, sensing, biomedicine, pharmaceuticals, cosmetics, oil recovery, emulsion polymerization, and heterogeneous catalysis, to site a few. The open literature demonstrates the ambitions to further develop this kind of emulsions with new functionalities and more complex structures, as well as exploring new applications. Thus, only a few examples are given here. Polymerization occurring in the Pickering emulsion systems has received a growing interest as revealed by several research activities (Glasing et al., 2019). Werner et al. have developed an appropriate approach to produce surfactant-free micro- and nanolatexes from Pickering emulsions stabilized by modified CNCs, which are grafted by acetyl moieties at the surface. The polymerization of the systems in the presence of thermoactive initiator produced a latex constituted by a mixture of polystyrene micro- and nanobeads, which were easily separated by filtration and centrifugation. The obtained beads can be used as reinforcement agents (Werner et al., 2017). Recently, Capron's research group has performed work studying the polymerization details in systems of pristine CNCs stabilized monomer (butyl methacrylate, lauryl methacrylate, styrene, etc.) droplets. This group produced latex particles with different sizes through two concomitant mechanism. Microparticles were produced by suspension polymerization mechanism, whereas the nanoparticles were obtained by emulsion polymerization mechanism (Saelices et al., 2019). In another work, Hérogues et al., investigated the Pickering emulsions of monomers stabilized with grafted CNCs by isobutyrate bromide moieties. It is revealed that such stabilizer can influence the nature of emulsion either direct (O/W), inverse (W/O), or double. The nature of emulsions led to produce diverse kinds of products, such as open-cell solids, beads, and capsules (Werner et al., 2018a). In a separate research work, Gao et al. have modified CNCs reducing end stabilizer and employed it during the polymerization. Polystyrene microspheres covered with asymmetric modified CNCs are generated (Du W. et al., 2017). CNC/polymer nanocomposites can be produced by a simple and environmentally friendly media, in which CNCs work both as stabilizer and as nanofillers in the emulsion and polymer compote. Réroguez's research group published an easy method to prepare polymer composites via polymerizations in Pickering emulsions stabilized by acetylated CNCs (Werner et al., 2018b). These authors reported that such method improved the mechanical features of the composite compared to the unfilled polystyrene sample. Similar enhancement of mechanical features is obtained for the poly(n-butyl methacrylate) composites. In recent years, Pickering emulsion based delivery systems have been used for encapsulation and controlled release applications in various fields such as cosmetic, food, biomedical, and pharmaceutics (Tang J. et al., 2019). For instance, Mackie et al. have produced sunflower (O/W) emulsions stabilized by CNCs, which were exposed to stimulated upper gastrointestinal tract digestion (Mackie et al., 2019). They demonstrated that CNCs were entrapped in the intestinal mucus layer and failed to reach the underlying epithelium, leading to the decrease of the adsorption of saturated lipids, thus concluded the effectiveness of CNC emulsifier through the reduction of plasma cholesterol. As an emerging application, the stability of CNC-based Pickering emulsion systems can be instantaneously controlled via external triggers by incorporating stimuli-responsive features, which can provide an appropriate platform to developing particular materials with outstanding properties. Tang et al. have recently grafted some polymers such as poly(methacrylic acid) onto CNCs surface (Tang et al., 2016). The authors claimed that the modified nanoparticles showed thermal and pH-responsive features. They revealed that the combination of stimuli-responsive properties with CNCs offer an easy and efficient way for oil harvesting applications. More recently, Li et al. have developed a new green and recyclable emulsifier for pH-responsive Pickering emulsion through the modification of CNCs with benzyl-polyethyleneimine (Ben-PEI-CNC) via the periodate oxidation of CNCs and reductive amination (Li W. et al., 2020). The authors demonstrated that the obtained Pickering emulsions stabilized by Ben-PEI-CNC are very sensitive to pH change, where the transition from stable emulsion to an unstable emulsion can be easily carried out. The authors claimed that this approach could open up new avenues for heterogeneous catalysis, emulsion polymerization, and oil recovery. Some other emerging applications based on Pickering emulsions stabilized by CNCs can also be find in the open literature such as antimicrobial applications, personal care products and cosmetic, improved food storage, composites with improved barrier characteristics and thermo-regulating materials (Fujisawa et al., 2017; Capron, 2018; Tang J. et al., 2019).

## Nanocellulose in Wood Adhesives

There are few reports which studied on the application of nanocellulose in wood adhesives (Lengowski et al., 2019; Vineeth et al., 2019). Commonly, wood adhesives can be classified into two big groups, which are soft and brittle adhesive. Isocyanate containing adhesives and polyethylene-vinylacetate including epoxies are less stiffer than amino-based and phenolic-based adhesives (Stoeckel et al., 2013). Good bonding within lignocellulosic part requires the understanding of complexity of wood polymer adhesive bonds in terms of surface chemistry, surface geometry, and adhesive penetration which the adhesive have to fulfill the ability of wetting to the wood surface in liquid state and the possibility to construct adequate cohesion within polymer during the curing state. The addition of high-stiffness and high-surface area of nanocellulose to such soft polymers explicitly results in stiffening of the cured modified adhesive. Those related to the fact that the cellulose addition rapidly increased the viscosity of the adhesive, therefore, limit the amount of nanocellulose that can be added into the system (Veigel et al., 2012) and affect the formation of bond line thickness as the nanocellulose also fills the hole and irregularities in the wood surface, which decreasing the porosity (Ayrilmis et al., 2016). Table 6 summarizes the effect of nanocellulose addition on the wood adhesive characteristics. The questions that need to be elaborated in the application of nanocellulose in wood adhesive are the mechanisms that responsible for the toughening effect and the study regarding the bond line structure of reinforced adhesive. Since urea-formaldehyde (UF) adhesive is the most common adhesive used in the wood-based composite industry, this section approaches to understand those mechanism in the scope of UF adhesive. An excellent review on UF adhesive resins for wood is reported in the literature (Dunky, 1998).

**Table 6:** The summarize of nanocellulose for wood adhesive applications reported in the literature.

**Nanocellulose form**	**Percentage cellulose addition (wt. %)**	**Resin**	**Findings**	**Reference**
CNF	Up to 1.33	Water based polyvinyl acetate latex-PVAc	Improved rheological behavior and bonding properties.	Richter et al., 2009
	Up to 10	One-component polyurethane (1C-PUR)		
CNC	Up to 2	UF	The formaldehyde emission of the UF resin decreased at optimum condition by adding only 1 wt. % Sulfuric acid hydrolysis (CNC).	Zhang et al., 2013
CNC	Up to 10	Hydroxypropyl cellulose	The employment of CNC is very promising in consolidation of wood without negative effect on its properties even after aging.	Hamed and Hassan, 2019
CNC	Up to 10	cottonseed protein	The introduction of CNC improves strength by 16% with respect to pure protein. The hot water resistance of cottonseed protein is also enhanced.	Cheng et al., 2019
CNC	Up to 5	UF	The incorporation of CNC in UF increased the liquid suspension viscosity, and the specimens exhibited a higher mechanical performance.	de Almeida Mesquita et al., 2018
CNC	Up to 3	PVAc	Modified PVAc showed higher bond strength at dry and wet observation and at elevated temperature.	Kaboorani et al., 2012
TEMPO-CNF	Up to 2	UF	UF-adhesive bonds can be significantly toughened by only small portion addition of nanocellulose.	Veigel et al., 2011
1MFC	Up to 5	UF at different F/U mol rations	The incorporation of MFC decreased the stress concentrations along the bond line, improved ductility of the adhesive.	Ayrilmis et al., 2016
MFC	0.5 to 5	UF	The fracture observation showed that the failure occurred in the wood rather than in the adhesive indicated the strength of the bond line by addition of MFC.	Kwon et al., 2015
MFC	Up to 5	UF	The addition of MFC indicated lower thermal stability at different F/U mol ratio except the enhancement of thermal stability at low F/U mol ratio which is 0.9 (E0).	Nuryawan et al., 2017

In addition, according to Richter et al. (2009), some critical parameter affecting the mechanical performance of the reinforced wood adhesive are the quality of controlled fibril morphology, the homogenous as well as the comparable polarity of fibrils and adhesive. In that case, methods of extraction and surface modification of nanocellulose dictate the behavior of modified adhesive and affect the bonding strength within adhesive and wood. Microfibrillated cellulose (MFC), which produced by high mechanical shearing fibrillation resulted in non-charged longer fibrillated cellulose lead high viscosity due to an entangled network structure in the adhesive (Veigel et al., 2012; Ayrilmis et al., 2016). TEMPO [(2,2,6,6-tetramthylpiperidin-1-yl)oxyl]-mediated cellulose oxidation (TEMPO-CNF), a longer fibrillated negative surface charged promote well-dispersion in polar adhesive (Isogai et al., 2011). While cellulose nanocrystals (CNC) modified with aminopropyltriethoxysilane (APTES) lowering the surface energy of modified CNC by increased about 26.4% on the contact angle between CNC and UF resin adhesive (Zhang et al., 2013). In other words, tunable wettability of reinforced adhesive could be obtained by modifying the surface of nanocellulose in order to manage the energy dissipation at the toughening mechanism of adhesive. It seem that the morphology of CNF has higher potential rather than that of cellulose whisker (CNC) to form a stable network and higher reinforcement efficiency, however, the amount of typical nanocellulose added should be sprayable for manufacturing.

Local stress concentration along the wood bond line in the brittle adhesives such as UF, phenolic formaldehyde (PF) and melamine-UF (MUF) is high due to high methylene crosslink density (Lubis et al., 2018) and the formation of crystalline region. Fortunately, there is possible interaction between hydroxyl groups of cellulose and methylol groups from UF resin that results in improvement of ductility of the adhesive (Fornué et al., 2011). The formation of various morphologies and size of crystalline region regarded as polycrystals in the UF resin adhesives especially at low formaldehyde/urea (F/U), i.e., 0.9 to 1.0 had been reported by Park and Causin (2013); Singh A. P. et al. (2014), and Nuryawan et al. (2017). Singh et al. studied the pattern of cured UF resin in contact with and without wood (Singh A. P. et al., 2014). The result suggested that at low formaldehyde thermosetting resin, in the presence of wood, cured UF resin possesses the distinct crystalline structure, although all the aspects from their study were poorly understood and still needed further clarification. The presence of the crystal was characterized under small angle X-ray scattering (SAXS) (Park and Causin, 2013). They believed that the crystalline regions took responsible for the stablility to hydrolysis and less hazardous due to the small release of formaldehyde gases. On the other hand, the addition of modified CNC had proof to reduce formaldehyde emission by 13% at optimum addition of 1 wt.% CNC as reported by Zhang et al. (2013). As far as author knowledge, there is no report, which studied the crystalline structure of UF resin in the presence of nanocellulose as reinforcing agent in adhesive, yet.

The main challenge of modified adhesive with nanocellulose is the adding either redispersible dried nanocellulose or nanocellulose suspension at the beginning of the adhesive synthesis (Gindl-Altmutter and Veigel, 2014). Simply, nanocelluloses were well-dispersed in a high concentrated formaldehyde solution (or dried formaldehyde), which was subsequently used for the synthesis of UF resin. Up to now, the cellulose-adhesive mixtures were commonly produced by adding aqueous nanocellulose into a commercial adhesive. Those method caused the limit addition of nanocellulose into adhesive. The more the cellulose suspension was added, the higher amount of water content in the system resulted in lowering the solid adhesive content which further slower the curing and gelling process (Kwon et al., 2015). The synthesis reaction of UF resin are possible to be conducted at three condition alkaline, weak acid, and strong acid. In this idea, the type of nanocellulose that used during synthesis would be limited to positive or uncharged nanocellulose for strong acid environment, since TEMPO-CNF with pKa ~3.50 will be agglomerated to form hydrogen bonding themselves and reduced dispersion in the matrix. Commercially, UF was synthesized with alkaline catalyst to initiate addition reaction and then was converted to acid side to promote the condensation reaction (down to pH 4.6) and then normalized to pH 8 to terminate the reaction (Nuryawan et al., 2017). More recently, Vineeth et al. demonstrated that the incorporation of NC in wood adhesives and crosslinking with binders such as poly (vinyl alcohol) may increase the performance and mechanical features, opening new possibilities for eco-friendly and bio-based wood adhesives (Vineeth et al., 2019). These achievements can reduce the dependency of petrochemicals in wood adhesives field in the near future.

## Nanocellulose for Adsorption, Separation, Decomtamination, and Filtration

In addition to the plethora of applications outlined above, extensive research activities continue to be conducted worldwide to conquer new domains and develop new sectors of uses of Nanocellulose.

NC materials have garnered much spotlight for application in the field of water treatment (Jamshaid et al., 2017; Mohamed et al., 2017b; Putro et al., 2017), dye removal (Karim et al., 2014; Mohammed et al., 2015), air purification (Gebald et al., 2011; Nemoto et al., 2015), and microbe and viruses decontamination (Wang et al., 2013; Rosilo et al., 2014; Li et al., 2018). In general, the mechanism for adsorption and separation is classified in four categories which are physical, chemical, biological, and acoustical, radiation and electrical processes (Mohamed et al., 2017b). The use of functionalized nanocellulose through sulfuric acid hydrolysis (SO^3−^), carboxylated groups (COO^−^), and amine (-NH_2_) groups ionically or covalently are used to select targeted contaminant or dyes (Gebald et al., 2011; Mohamed et al., 2017a). Immobilized mycelia *Pestalotiopsis* sp NG007 showed the ability to grow and decolorized some reactive dyes due to the laccase enzyme activities (Yanto et al., 2014). Further, laccase immobilization on nanocellulose fibrils by electrospinning process has been reported by Sathishkumar et al. (2014). They proposed an eco-friendly system that may be used to treat textile effluent which contains a mixture of different dyes and salts. Laccase immobilization on nanocellulose also can be used as antimicrobial membrane for wound dressing applications (Sampaio et al., 2016).

Nanocellulose composites have been used for heavy metal removal in water environment. Heavy metals such as Zn^2+^, Cu^2+^, Cd^2+^, Hg^+^, Pb^2+^, Cr^3+^ are known as toxic and possible to accumulate in living organism and human body. Wang et al. produced composite membrane of polyacrylonitrile (PAN)/microscale polyethylene terephthalate (PET) fibrous scaffold with 5 nm cellulose nanofibrils (Wang et al., 2013). The high surface area, fibrous structure and high porosity of nanocellulose induced high adsorption of Cr^6+^ and Pb^2+^ up to 100 and 260 mg/g, respectively. Meanwhile, Yu et al. reported the modified cellulose nanocrystals with succinic anhydride could improve the adsorption rate up to 465.1 mg/g which is the highest adsorption rate of heavy metals have been reported so far (Yu et al., 2013). An excellent review on nanocellulose as novel nanostructured for environmental remediation was presented by Mahfoudhi and Boufi (2017).

Nanocellulose appeared as adsorption in common applications in the form of cellulose beads composite with others organic materials such as chitin (d'Halluin et al., 2017), chitosan (Li and Bai, 2005), or sodium alginate (Vijayalakshmi et al., 2016), composite with inorganic compounds such as sodium montmorillonite (NaMMT) (Kumar et al., 2012), titanium dioxide (TiO_2_) (Li et al., 2015) or ferric chloride solution (Kim et al., 2006), hydrogels (Jamshaid et al., 2017), and aerogels form (Wei et al., 2018, 2019; Gu et al., 2020). Recently, Tchikovhi et al. Have comprehensively reviewed the nanocellulose based composites reinforced with activated carbon, carbon nanotube, graphene oxides, metal, non-metals, and ceramics as adsorbents for diverse organic and inorganic contaminants in water (Tshikovhi et al., 2020). They also reported the eventual interactions between adsorbent and adsorbates, which can influence the efficiency of the adsorption process. It is demonstrated that the most of the adsorption process involves interactions between the pollutants and the materials through different mechanisms such as electrostatic interaction, van der Waals forces, hydrogen bondings, and pi-pi interactions.

Recyclable cellulose nanocrystal reinforced alginate hydrogels have been reported by Mohammed et al. (2015). Methylene blue (MB) is used as adsorption-desorption model which the removal efficiency of MB remained at 97% after five time cycles. The adsorption rate is influenced by contact time, initial dye concentration, pH, temperature, ionic strength, crosslinking density, and bead size. Thermodynamic adsorption study such as free energy (ΔG°), enthalpy (ΔH°), and entropy (ΔS°) could be calculated from the effect of temperature vs. percentage of dye removal. A highly recyclable cellulose beads, 10 times adsorption-desorption with 86.83% efficiency, for highly efficient dye removal 288.81 mg/g adsorption capacity was obtained by highly carboxylated cellulose (4.93 mmol/g) (Meng et al., 2019). They produced highly carboxylated cellulose by introduced citric acid in the presence of trisodium citrate catalysts and reacted dissolved cellulose for 2–6 h at 110–120°C. On the other hand, highly carboxyl cellulose nanofibrils with no change in the crystal structure, higher molar-mass, and better thermal stability could be produced by esterification reaction using maleic anhydride (Iwamoto and Endo, 2015) and succinic anhydride (Sehaqui et al., 2017). Those highly carboxylated CNF were effectively integrated into paper filters for removal lead from aqueous solution or as transparent films for advanced applications.

NC-based materials as efficient adsorbent and flexible membrane have been recently reviewed by Abouzeid et al. revealing that such materials with outstanding features such as high surface area, better mechanical characteristics, hydrophilicity, and tailorability of the surface chemistry through grafting anionic and cationic surface chemical groups match with the prerequisites for wastewater treatment materials (Abouzeid et al., 2018). More recently, a comprehensive review article has been published by Köse et al., dealing with NC-based adsorbents, revealed the importance of such new materials as viable sustainable alternatives as adsorbents (Köse et al., 2020). In another work, Sharma et al. have revealed that nanocellulose is become an important, safe, and economically sensible new material that is particularly appropriate for membrane applications (Sharma et al., 2020). They reported that nanocellulose membranes developed from inexpensive, abundant, and sustainable resources, such as agricultural residues and underutilized biomass waste, can lower the cost of membrane separation, as these membranes will offer the ability to remove a range of pollutants in one step, via size exclusion and/or adsorption. The nanocellulose-enabled membrane technology not only may be suitable for tackling global drinking water challenges, but it can also provide a new low-cost platform for various pressure-driven filtration techniques, such as microfiltration, ultrafiltration, nanofiltration, and reverse osmosis. However, further research activities need to be conducted in the future to reduce cost of processing with improving performance parameters.

## Conclusions and Future Perspectives

The present review reports advances in the preparation, modification, and employment of nanocellulose, especially cellulose nanocrystals, as principal ingredients for various emerging applications. It provides knowledge to stimulate further research works in this field. Although the literature of nanocellulose, produced from several natural occurring sources using different approaches, has been intensively studied over the past 20 years, some challenges should be overcome, particularly in the fields of surface and end-reducing modifications, the improvement of environmentally-friendly processes of extraction at lower cost with reduced energy-consuming processes as well as the up-scaling production. It is demonstrated in the present review that nanocellulose shows the potential to be truly green nanomaterial with several outstanding useful features such as high surface area, tailorability of surface chemistry, better mechanical characteristics, anisotropic shape, among others, making it an excellent material for widespread range of applications in the field of biomedical engineering and material science, and it exhibits a high potential for evolving industries. With the emergence of cost-effective commercial sources of nanocellulose, a room for new applications and improvement of the existing ones, which can be employed in various industries that require materials with advanced properties, still exists and such topic is of particular interest for the future. Therefore, further research activities need to be conducted to fill current gaps through the practical transition from laboratory scale to industrial or commercial production, and achieve the feasibility of the final materials and introduce them in the market, in particular, (1) optimize the whole process and develop new methods to produce new NC-based materials; (2) use of life cycle assessment to some environmental aspects of NC-based materials; (3) decrease the energy- and time-consumption of the NC-based materials. Despite the above-mentioned challenged, we expect that nanocellulose-based materials will certainly improve the people's quality of life in the future through the development of the next generation of materials.

## Author Contributions

DT and MH took the lead in writing the manuscript. All authors provided critical feedback and helped shape the research, analysis, and manuscript.

## Conflict of Interest

The authors declare that the research was conducted in the absence of any commercial or financial relationships that could be construed as a potential conflict of interest.
